# Four Transformer-Based Deep Learning Classifiers Embedded with an Attention U-Net-Based Lung Segmenter and Layer-Wise Relevance Propagation-Based Heatmaps for COVID-19 X-ray Scans

**DOI:** 10.3390/diagnostics14141534

**Published:** 2024-07-16

**Authors:** Siddharth Gupta, Arun K. Dubey, Rajesh Singh, Mannudeep K. Kalra, Ajith Abraham, Vandana Kumari, John R. Laird, Mustafa Al-Maini, Neha Gupta, Inder Singh, Klaudija Viskovic, Luca Saba, Jasjit S. Suri

**Affiliations:** 1 Department of Computer Science and Engineering, Bharati Vidyapeeth’s College of Engineering, New Delhi 110063, India; siddgupta462@gmail.com; 2 Department of Information Technology, Bharati Vidyapeeth’s College of Engineering, New Delhi 110063, India; arudubey@gmail.com (A.K.D.); neha06gupta@gmail.com (N.G.); 3 Department of Research and Innovation, Uttaranchal Institute of Technology, Uttaranchal University, Dehradun 248007, India; drrajeshsingh004@gmail.com; 4 Department of Radiology, Massachusetts General Hospital, 55 Fruit Street, Boston, MA 02114, USA; mkalra@mgh.harvard.edu; 5 Department of Computer Science, Bennett University, Greater Noida 201310, India; ajith.abraham@bennett.edu.in; 6 School of Computer Science and Engineering, Galgotias University, Greater Noida 201310, India; vandana.soni80@gmail.com; 7 Heart and Vascular Institute, Adventist Health St. Helena, St. Helena, CA 94574, USA; lairdjr@ah.org; 8 Allergy, Clinical Immunology and Rheumatology Institute, Toronto, ON M5G 1N8, Canada; dralmainioffice@gmail.com; 9 Department of Radiology and Ultrasound, University Hospital for Infectious Diseases, 10000 Zagreb, Croatia; drindersingh1@gmail.com; 10 Department of Radiology, Azienda Ospedaliero Universitaria (A.O.U.), 09100 Cagliari, Italy; klaudija.viskovic@bfm.hr; 11 Department of ECE, Idaho State University, Pocatello, ID 83209, USA; lucasabamd@gmail.com; 12 Stroke Diagnostics and Monitoring Division, AtheroPoint™, Roseville, CA 95661, USA; 13 Department of Computer Engineering, Graphic Era (Deemed to be University), Dehradun 248002, India; 14 Department of Computer Science & Engineering, Symbiosis Institute of Technology, Nagpur Campus 440008, Symbiosis International (Deemed University), Pune 412115, India

**Keywords:** chest X-ray, segmentation, attention mechanisms, vision transformers, convolutional neural networks, layer-wise relevance propagation

## Abstract

*Background*: Diagnosing lung diseases accurately is crucial for proper treatment. Convolutional neural networks (CNNs) have advanced medical image processing, but challenges remain in their accurate explainability and reliability. This study combines U-Net with attention and Vision Transformers (ViTs) to enhance lung disease segmentation and classification. We hypothesize that Attention U-Net will enhance segmentation accuracy and that ViTs will improve classification performance. The explainability methodologies will shed light on model decision-making processes, aiding in clinical acceptance. *Methodology*: A comparative approach was used to evaluate deep learning models for segmenting and classifying lung illnesses using chest X-rays. The Attention U-Net model is used for segmentation, and architectures consisting of four CNNs and four ViTs were investigated for classification. Methods like Gradient-weighted Class Activation Mapping plus plus (Grad-CAM++) and Layer-wise Relevance Propagation (LRP) provide explainability by identifying crucial areas influencing model decisions. *Results*: The results support the conclusion that ViTs are outstanding in identifying lung disorders. Attention U-Net obtained a Dice Coefficient of 98.54% and a Jaccard Index of 97.12%. ViTs outperformed CNNs in classification tasks by 9.26%, reaching an accuracy of 98.52% with MobileViT. An 8.3% increase in accuracy was seen while moving from raw data classification to segmented image classification. Techniques like Grad-CAM++ and LRP provided insights into the decision-making processes of the models. *Conclusions*: This study highlights the benefits of integrating Attention U-Net and ViTs for analyzing lung diseases, demonstrating their importance in clinical settings. Emphasizing explainability clarifies deep learning processes, enhancing confidence in AI solutions and perhaps enhancing clinical acceptance for improved healthcare results.

## 1. Introduction

The global onslaught of lung diseases, notably bacterial pneumonia, viral pneumonia, tuberculosis, and the recent COVID-19 pandemic, has starkly highlighted the limitations of current healthcare systems in managing such conditions effectively. The mortality rates associated with these diseases, compounded by the emergence of COVID-19 [1,2,3], have not only resulted in a significant loss of life but have also imposed considerable economic burdens worldwide. The pandemic has exacerbated the complexities of lung disease diagnosis and management, especially in patients with co-morbidities like diabetes and neurological disorders [4,5], which can dramatically increase the severity of COVID-19. The resulting condition, often referred to as long COVID [6], is characterized by a prolonged battle between the body’s immune response, including elevated levels of inflammatory markers such as Interleukin-6 (IL-6) and Tumor necrosis factor-alpha (TNF-alpha), as well as the virus [7]. This ongoing conflict weakens the immune system, making the body more susceptible to environmental factors and further complicating lung conditions.

Chest computed tomography (CT) scans are important for thoroughly evaluating lung diseases by providing comprehensive high-resolution pictures that help accurately identify anatomical structures and characterize diseases. Yet, their effectiveness is greatly hindered by their expensive operational costs, complex infrastructure needs, and the necessity for specialized technical skills [8]. These factors combined result in their scarce availability and restricted access, particularly in remote and resource-limited areas [9]. This gap highlights the pressing need for diagnostic approaches that are more economically feasible and widely accessible. Chest radiographs, or chest X-rays (CXRs), are a more practical choice since they are less expensive, need fewer resources, and are more widely accessible in various healthcare settings. However, CXRs present specific difficulties, particularly due to the tendency for image noise [10], which may obstruct important diagnostic information and make the interpretation process more complex. Moreover, depending on human experience to analyze these radiographs increases subjectivity, which might result in variability in diagnosis results. This variation is due to variations in observer expertise, perceptual understanding, and cognitive exhaustion, which can impact the accuracy and reliability of diagnoses made from CXR assessments. The intersection of these factors complicates the diagnostic process and emphasizes the need for new methods to reduce these restrictions, improving the accuracy and accessibility of diagnosing and managing pulmonary diseases.

In response to these pressing challenges, we propose an innovative, low-cost artificial intelligence (AI)-driven diagnostic system that leverages deep learning (DL) technologies to automate the analysis of CXRs. This system aims to overcome the limitations of manual interpretation by utilizing advanced algorithms for lung segmentation and disease classification, thereby enhancing the accuracy and efficiency of lung disease diagnosis. Our approach employs a diverse array of models, combining the strengths of traditional convolutional neural networks (CNNs) with cutting-edge architectures. Specifically, we utilize ResNet 50 [11] and Visual Geometry Group 16 (VGG 16) [12] for their DL capabilities in feature extraction, EfficientNet-B7 [13] for its scalability and efficiency in handling complex image data, and Inception V3 [14] for its inception modules that allow for a wider network with fewer parameters. Alongside these, we incorporate Vision Transformers (ViTs) [15] for their ability to capture global context, ViT Large for its enhanced capacity in processing detailed image features, and DeiT [16] (Data-efficient Image Transformers) for its efficiency in training with fewer data. Additionally, we explore hybrid models such as MobileViT [17], which combines the perceptual benefits of ViTs with the efficiency of CNNs, offering a comprehensive solution for detailed image analysis and robust disease classification in lung diseases.

The proposed study focuses on the premise that incorporating ViTs with self-attention processes will provide better diagnostic accuracy than conventional CNN models. ViTs have the ability to evaluate pictures as sequences of patches, which allows for a detailed comprehension of specific aspects important for precise illness categorization. We have created a comprehensive experimental protocol to validate our approach. This includes comparing model performance with and without lung segmentation, examining attention mechanisms, and conducting thorough statistical analysis with methods like Tukey’s Honestly Significant Difference and McNemar test, as well as many more statistical tests. The utilization of explainability methods such as Grad-CAM++ [18,19,20] and Layer-wise Relevance Propagation (LRP) [21,22,23] will offer vital insights into the decision-making mechanisms of our models, clarifying the AI “black box” and building trust in AI-based diagnostics. By bridging the gap between advanced AI technologies and clinical practice, our system not only aims to improve the diagnostic accuracy and efficiency of lung disease detection but also to make these advancements accessible and understandable to clinicians and patients alike. This initiative represents a significant step forward in the utilization of AI in healthcare, promising to revolutionize the diagnosis of lung diseases and ultimately enhance patient outcomes.

This paper explores the transformative potential of AI in diagnosing lung diseases, starting with an introduction in Section 1 that underscores the increasing prevalence of these illnesses and the urgent need for more advanced diagnostic tools. Section 2 reviews traditional diagnostic methodologies and introduces AI’s role in revolutionizing medical imaging, highlighting the limitations of existing methods alongside the capabilities of ViTs. The methodology in Section 3 describes a detailed DL pipeline employing CNNs, ViTs, hybrid models, and Attention U-Net for lung disease analysis. This section outlines the experimental setups, protocols, loss functions, model assessment metrics, and optimization strategies used, enhancing the study’s transparency and reproducibility. The results in Section 4 offer a thorough assessment of the models’ performances, focusing on the capabilities of the Attention U-Net and ViTs. Section 5 assesses model performance evaluation, using Receiver Operating Characteristic (ROC) curves and Area Under the Curve (AUC) metrics to gauge the models’ reliability and precision. A comparative analysis in Section 6 examines differences in performance between ViTs and traditional CNNs, contrasts U-Net with Attention U-Net, and evaluates classification effectiveness on raw versus quality-controlled data. Section 7 ensures the robustness and reliability of the findings with statistical and reliability analysis, employing Tukey’s Honestly Significant Difference test and the McNemar test for binary classification models. Section 8 addresses explainability, focusing on the theory and interpretation of results from methods like LRP and Grad-CAM++. A critical discussion in Section 9 synthesizes insights from various sections, summarizing key findings, benchmarking them against existing research, and discussing the unique advantages of ViTs. The conclusion in Section 10 summarizes the significant potential of AI to transform lung disease diagnostics, linking technological advancements to practical clinical applications and outlining future research directions in AI-driven diagnostics. This structure is designed to make the findings accessible and comprehensible, minimizing confusion while maximizing the impact of the analysis on real-world clinical practices.

## 2. Background

The history of lung disease detection has been marked by significant advancements, yet it has always faced challenges. Initially, CXRs and CT scans were the primary modalities used for diagnosing lung diseases. While chest CT scans provided high-resolution images essential for detailed anatomical evaluations, their high cost and the need for specialized technical expertise made them less accessible, particularly in resource-limited settings. Chest X-rays, although more affordable and widely available, were subject to image noise and variability in interpretation [24,25,26] making accurate diagnosis challenging and highly dependent on the radiologist’s expertise.

The advent of machine learning (ML) brought a positive shift in medical imaging [27,28], aiming to reduce subjectivity and improve diagnostic accuracy. Early ML applications focused on automating image analysis tasks, which showed promise in enhancing diagnostic procedures. However, the inherent limitations of CXRs, such as poor contrast and difficulty in extracting borders, posed significant challenges. These issues often led to inconsistent results, thereby limiting the clinical success of ML-based approaches in lung disease diagnosis [29]. The evolution of DL marked a pivotal moment in the field of medical imaging [30,31,32,33,34]. CNNs emerged as powerful tools for image segmentation and classification [35,36,37,38]. CNNs, with their ability to automatically learn hierarchical feature representations from data, significantly improved the accuracy of lung disease detection from medical images. They were particularly effective in tasks like nodule detection, pneumonia classification, and tuberculosis screening, outperforming traditional ML methods [39,40,41,42,43,44,45]. Despite their success, CNNs had limitations in capturing global contextual information, which sometimes affected their performance in complex image analysis tasks.

To address the limitations of CNNs, ViTs were introduced. ViTs leverage self-attention mechanisms to analyze images as sequences of patches, similar to how natural language processing models handle text. This approach allows ViTs to capture global contextual information more effectively than CNNs. ViTs have shown promise in generating detailed and interpretable heatmaps for lesion detection and segmentation, thereby enhancing diagnostic accuracy. Their ability to provide a holistic view of the image makes them particularly suitable for complex medical imaging tasks. Explainability in AI diagnostics is vital for transparency, with methods like Grad-CAM++ and LRP providing visual insights into model predictions, enhancing trust and clinical acceptance. Despite progress in AI for lung disease diagnosis, gaps remain in integrating CNNs, ViTs, and advanced explainability methods. This study addresses these gaps through comprehensive model comparisons, explainability techniques, and rigorous statistical evaluations, aiming to improve diagnostic accuracy and efficiency.

## 3. Methodology

**Figure 1:**
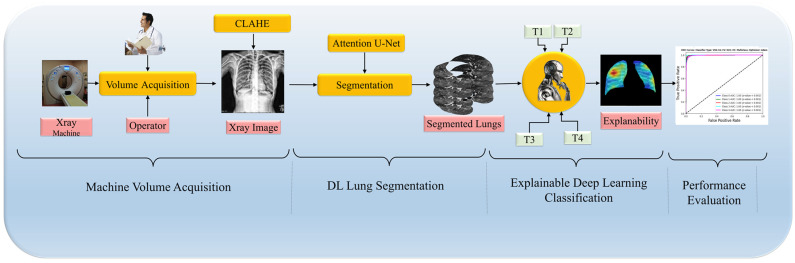
The DL pipeline for lung pathology analysis, from X-ray acquisition to explainable Transformer-based classification; here, T1, T2, T3, and T4 represent ViTs.

In this paper, we introduce a DL pipeline tailored for enhancing the accuracy of medical imaging diagnostics, particularly in detecting lung diseases through CXR, illustrated in Figure 1. The pipeline begins with rigorous data curation, which includes preprocessing and applying Contrast Limited Adaptive Histogram Equalization (CLAHE), ensuring that models like ViTs and CNNs are robust and generalizable. Next, we employ the Attention U-Net model for lung region segmentation, critical for accurately localizing pathology. This is followed by a classification phase where the unique strengths of ViTs and CNNs are harnessed to differentiate between various lung disease manifestations, aiming to boost classification accuracy. The pipeline concludes with an explainability assessment to clarify the decision-making processes of the models by analyzing generated heatmaps, ensuring the interpretability of the findings. A rigorous set of experimental protocols with comprehensive metrics and statistical analysis supports this methodology, demonstrating that ViTs can enhance explainability without sacrificing segmentation or classification quality. This integrative approach marks a significant step forward in leveraging DL for improved clinical decision making in radiology. Furthermore, our framework’s adaptability allows for the future integration of additional diagnostic modalities, expanding its utility in clinical practice.

### 3.1. COVID-19 Data

**Figure 2:**
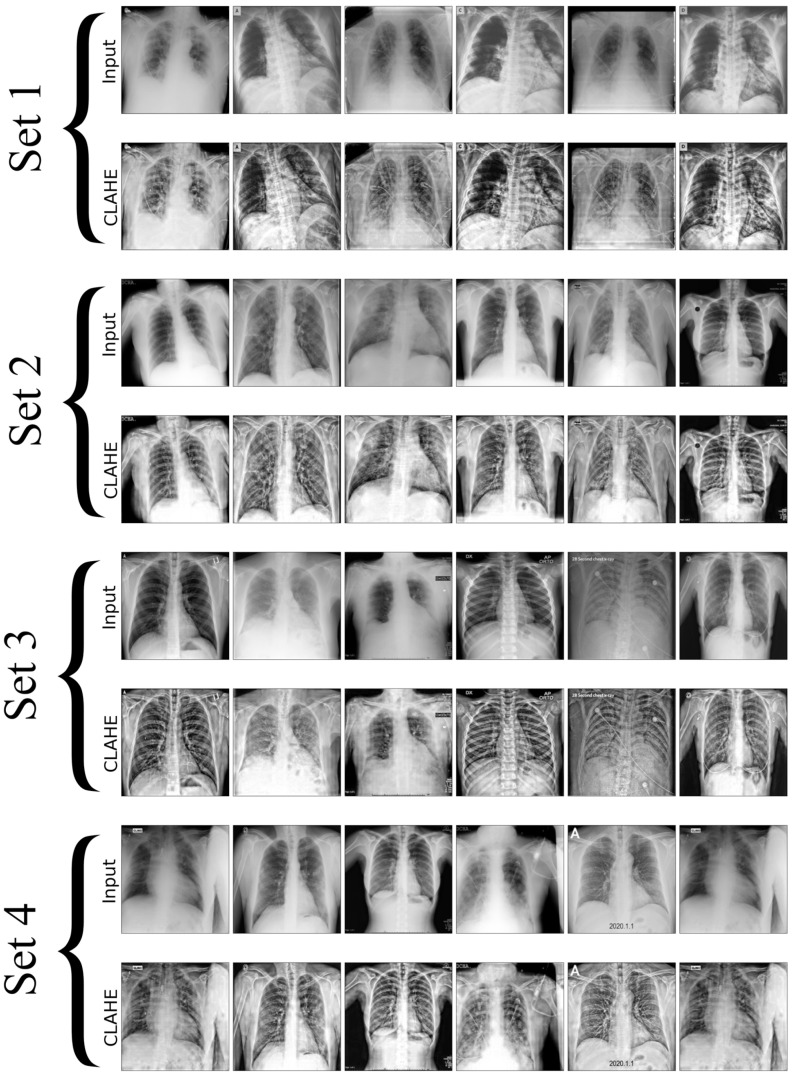
Side-by-side comparison of a chest X-ray before and after CLAHE enhancement.

In this research, we utilize a meticulously curated CXR dataset with 12,000 images encompassing a spectrum of pulmonary conditions, including normal cases with no apparent pulmonary pathology, bacterial pneumonia characterized by bacterial infections, viral pneumonia indicating viral infections, tuberculosis with radiographic signs typical of TB, and COVID-19 marked by features such as ground-glass opacities and consolidation, as shown in Figure 2. Each set in the image contains unique chest X-rays and consists of two rows: the first row shows the original input images, and the second row displays the images processed with CLAHE. These images were sourced from three distinct Kaggle databases [46,47,48], each acknowledged for its specialized collection that contributes to the broad spectrum of medical conditions represented, where the input and resultant CLAHE-enhanced images are juxtaposed to illustrate the method’s impact. The CLAHE process is pivotal for augmenting image contrast, which in turn renders subtle pulmonary details more conspicuous, significantly improving the detectability and classification accuracy of lung diseases. The figure accentuates how CLAHE amplifies the visibility of lesions, underscoring its significance in our preprocessing regimen. Post-preprocessing, the dataset is partitioned into training, validation, and testing sets, ensuring an even representation of various lung pathologies across each subset to facilitate a balanced and comprehensive evaluation of the model’s performance. The meticulous preparation of datasets is essential for improving DL model’s capacity to generalize, from the initial data acquisition through to explainability and performance evaluation. This process significantly boosts the effectiveness and reliability of lung disease detection models.

### 3.2. Model Selection

We have used the Attention U-Net model [49] for segmentation tasks in our work, in addition to the previously described architectures. It is crucial to include the Integrating Attention U-Net, as it enhances the ability to concentrate on important aspects in CXRs by integrating attention processes. This method efficiently addresses segmentation difficulties by assigning different degrees of importance to different areas in the picture, resulting in more precise and accurate segmentation results. Attention processes allow the model to concentrate on certain regions of interest, such as anomalous tissue patterns or lesions that suggest lung illness, enhancing the accuracy of disease identification and analysis. This method is anticipated to alleviate the constraints encountered by conventional U-Net [50], particularly in scenarios where the areas of interest are faint or partly hidden. We will compare the conventional U-Net and Attention U-Net in the experimental technique part of our research. This work emphasizes the advantages of incorporating attention processes into segmentation models to enhance segmentation accuracy and prioritize key components in CXRs, thus enhancing the comprehension of lung disease characteristics. Choosing suitable categorization models is crucial for our study, as it directly influences the efficiency and reliability of our results. We use many models in our approach, including CNNs, ViTs, and hybrid models. Selection is divided into three independent groups to examine a range of deep learning architectures and assess their performance in diagnosing lung illnesses using CXR.

The first group comprises CNN models like VGG 16, ResNet 50, Inception V3, and EfficientNet B7. These models are well known for their ability to extract features effectively and have been crucial in advancing the field of image recognition. The second set focuses on ViTs, including ViT Base, ViT Large, and DeiT. Transformers are selected for their sophisticated self-attention mechanism, allowing for a thorough analysis of image data, which may be advantageous in identifying tiny patterns crucial for classifying lung diseases. The third type comprises hybrid models that combine the benefits of CNNs and ViTs to use both local feature extraction and global contextual understanding. This paper demonstrates the use of hybrid strategies in MobileViT to enhance model performance and interpretability. This study’s objective is to examine and contrast conventional and sophisticated DL models for the diagnosis of lung illnesses. We will assess segmentation accuracy, classification performance, and interpretability using heatmap visualization.

### 3.3. Architecture

#### 3.3.1. Segmentation

The Attention U-Net model is based on U-Net architecture, a customized DL model often used for segmenting medical pictures. The idea incorporates “attention gates” into the skip connections of the U-Net. The gates function as filters, emphasizing important characteristics while separating and reducing less important input. Improved segmentation accuracy highlights important portions of the image, assisting in pinpointing particular structures or anomalies in intricate medical imaging. The attention approach provides interpretability by showing the precise image areas that the model concentrated on to create its segmentation output.

**Figure 3:**
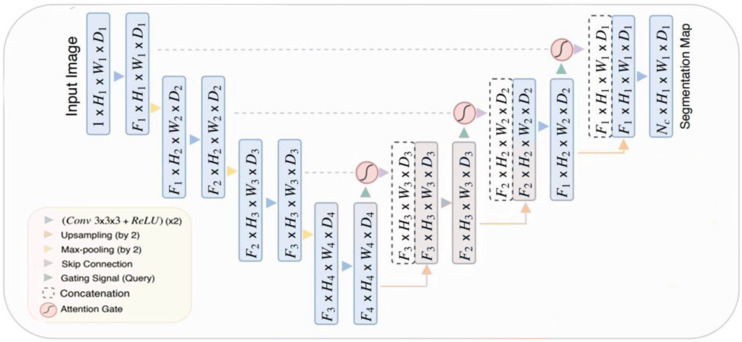
Architecture of Attention U-Net.

Figure 3 illustrates the Attention U-Net architecture, a customized version of the U-Net specifically created for medical image segmentation. The encoder route uses convolutions and downsampling to extract abstract features, while the decoder path uses upsampling and convolutions to restore resolution. Skip connections connect the encoder and decoder to guarantee accurate localization. The Attention U-Net model integrates attention gates into the skip connections. The gates alter the importance of characteristics, emphasizing crucial areas for division and reducing unnecessary information, resulting in improved segmentation precision.

#### 3.3.2. Classification of Vision Transformer Models

##### ViT-Base

The ViT model is a prime example of using Transformer structure for visual tasks. It utilizes the potent self-attention processes that have propelled the Transformer models to success. It considers image patches similar to tokens in a language model, resulting in a flattened two-dimensional representation that allows for attention-based contextual interaction among patches. It is essential to include the standard ViT to analyze the fundamental characteristics of Transformer-based image analysis, such as the performance advantages and interpretability provided by its self-attention layers.

**Figure 4:**
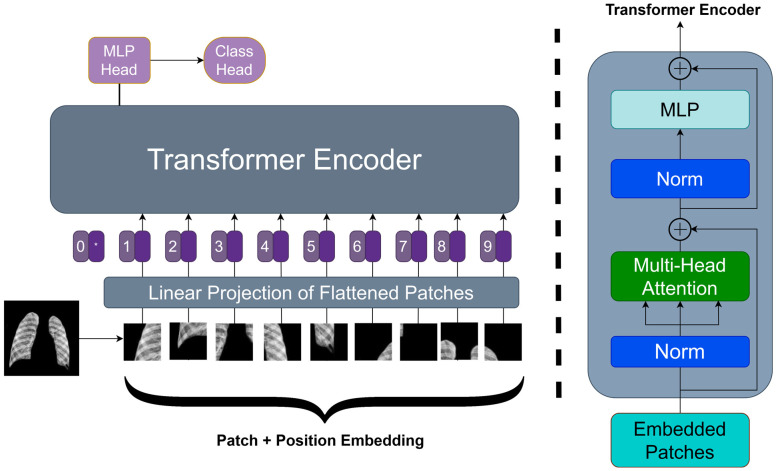
Architecture of Vision Transformer.

Figure 4 illustrates the basic architecture of a Vision Transformer (ViT-Base), a model that applies transformer principles, extensively used in natural language processing, for image recognition. The procedure begins by partitioning the input image into patches of a certain size and then mapping these patches to a lower-dimensional space via linear transformation. Positional embeddings are then used to maintain the spatial information of each patch. The embedded patches serve as the input sequence for the transformer encoder, including alternating layers of multi-head self-attention and multilayer perceptron (MLP) networks. Normalization layers follow each layer and include residual connections to facilitate gradient flow in training. The output of the transformer encoder is sent into a Multi-Layer Perceptron (MLP) head, sometimes known as the class head. The head consists of interconnected layers that provide categorization predictions. The ViT architecture, created by Dosovitskiy et al. [15], is known for its ability to capture long-range dependencies in images and adapt well to various model sizes, marking a significant advancement in computer vision.

##### ViT Large

We used the ViT-Large model to evaluate the impact of increased model sizes on performance and explainability. Increased parameters provide a more complex and comprehensive depiction, perhaps enhancing accuracy in image recognition. The ViT-Large variant is used as a framework to investigate the balance between performance and interpretability in bigger Transformer models.

Figure 4 illustrates the construction of the Vision Transformer (ViT-Base), a basic model for image classification tasks that applies transformer concepts. Figure 4 depicts the fundamental notion necessary for understanding the bigger and more elaborate version, the ViT-Large. Both systems share a fundamental principle of partitioning an input image into fixed-size patches, linearly projecting these patches into embeddings and including positional encodings to maintain spatial context. The ViT-Large design is distinguished only by its size. The transformer encoder is expanded with more layers and a wider network to handle complex patterns and support a greater number of parameters. The ViT-Large model has expanded the size of both the multi-head self-attention and MLP blocks, allowing for more extensive feature integration throughout the patches. The ViT-Large model often surpasses the ViT-Base model on benchmark datasets because of its enhanced capacity to grasp long-range correlations and intricate features in image data. Figure 4 shows the schematic of the ViT-Base model, highlighting conceptual and architectural features that may be directly applied to and form the basis for the more sophisticated ViT-Large model.

##### Data-Efficient Image Transformer

DeiT is a notable shift in the training approach of Transformer models, emphasizing the optimization of data efficiency without compromising performance. This paradigm addresses the need for extensive data by using techniques such as knowledge distillation. It is an advanced method for analyzing the connection between data efficiency, model complexity, and interpretability.

**Figure 5:**
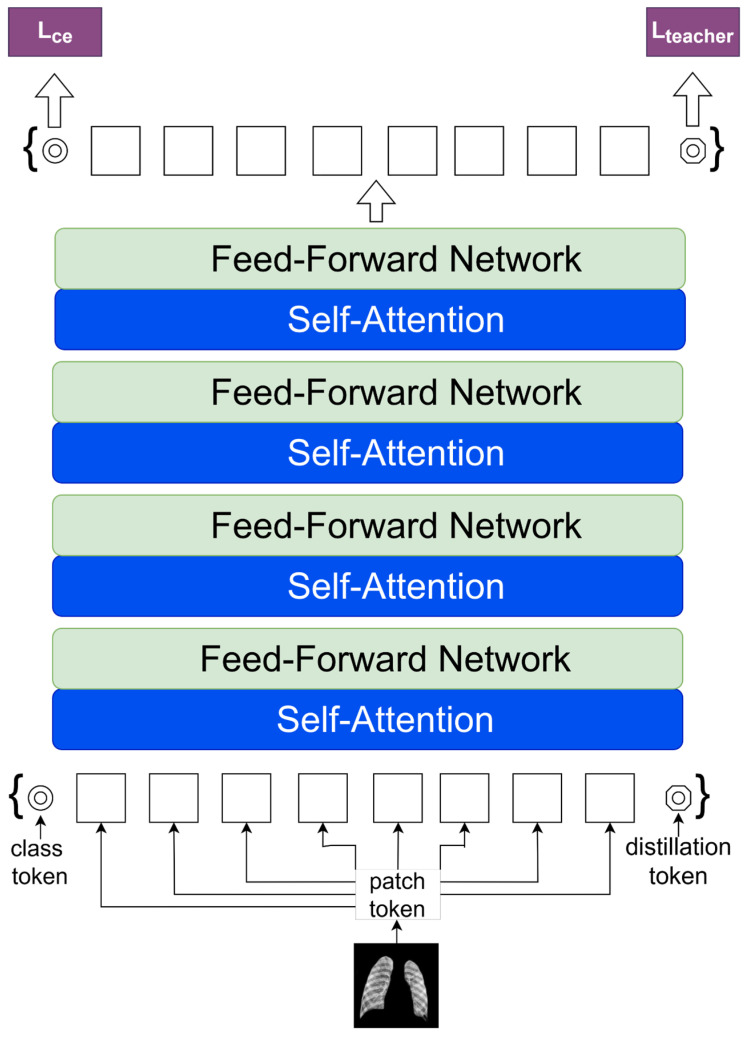
Architecture of Data-efficient Image Transformer model.

Figure 5 depicts the creation of a DeiT model. DeiT improves the ViT approach by adding a distillation token to the class token, enabling it to learn from a pre-trained instructor model without the need for extensive datasets. The input image is first segmented into patches and then converted into embeddings by linear projection, similar to the procedure in ViT. The embeddings, class token, and distillation token undergo processing in a sequence of blocks inside the transformer. Each block has a self-attention mechanism to determine the importance of patches and a feed-forward network to analyze patch embeddings. Layer normalization is applied before each component in the transformer encoder structure, followed by a residual connection after each. The class token gathers data from the image and is used for the final classification prediction (L_ce) in accordance with the standard procedure in ViT. The distillation token is a new addition designed to enhance the extraction of information from the teacher model (L_teacher). It is inspired by knowledge distillation, a process where a smaller student model mimics the behavior of a larger, more complex model. DeiT can effectively train with less datasets compared to ViT, which often requires large amounts of data to perform well. The inclusion of the distillation token in the DeiT model showcases the architecture’s unique approach to improving training data efficiency, addressing a key challenge in using transformers for computer vision tasks.

##### MobileViT

MobileViT is a balanced design that integrates Transformer and CNN techniques to enhance performance on mobile devices by reducing the computational load of traditional ViT models. This collection features sophisticated efforts to achieve a compromise between high accuracy and low processing complexity, enabling Transformer technology to be used on mobile and resource-constrained systems.

**Figure 6:**
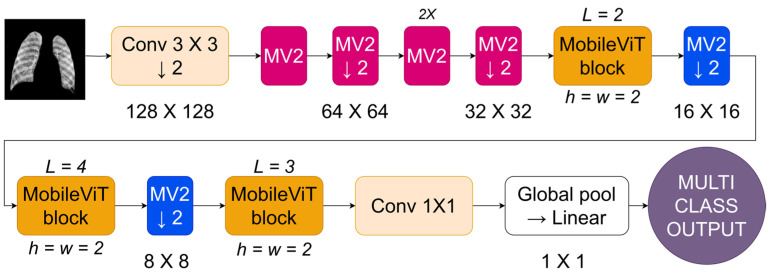
Architecture of MobileViT model.

Figure 6 depicts the MobileViT architecture, a CNN designed to enhance image processing on mobile devices. The network starts by inputting an image and using a standard convolutional layer (Conv 3 × 3) with a stride of 2. This reduces the resolution to 128 × 128 and captures low-level information. The approach utilizes MobileNetV2 (MV2) blocks with depthwise separable convolutions to reduce computational expenses while maintaining representational effectiveness beyond the first convolution. The MV2 blocks are used sequentially, with each block reducing the image’s resolution by a factor of 2, leading to a steady decrease in spatial size over the stages (64 × 64, then 32 × 32). In Figure 6, the forward arrow represents the sequence of operations, while the down arrow indicates downsampling, which reduces the image size. After each group of MV2 blocks, a MobileViT block is included. The blocks use transformer-based self-attention methods tailored for mobile applications by confining the self-attention to tiny windows (h = w = 2) rather than the whole feature map. The network’s tight focus allows it to capture important geographical links while effectively managing computer resources. The MobileViT blocks are structured hierarchically with different depths (L = 2, L = 4, L = 3) to allow the network to capture features at different scales.

The design merges the extracted features by using a 1 × 1 convolution to mix them and then uses a global pooling layer to consolidate the feature maps into a singular representation. The merged vector is then fed into a linear layer to obtain the final output, which is appropriate for tasks like image categorization. MobileViT combines depthwise separable convolutions with local self-attention to provide an efficient architecture for vision tasks on cellphones with limited resources.

##### Convolutional Models

In the methodology part of our study, choosing CNN models is crucial for a thorough comparison of image processing methods, focusing on their performance and ability to provide understandable insights. The selected models, 1, Inception V3, VGG 16, and EfficientNet B7, showcase several architectural philosophies in DL, each offering distinct methods for managing spatial hierarchies and extracting features. For an in-depth view of the architecture, see Appendix A. ResNet 50 is well known for its effective use of residual connections, which helps overcome the difficulty of training deeper networks by improving the gradient flow in backpropagation. This feature allows for the creation of a network that is deep and can learn complex abstract representations of input data, making it a crucial model for assessing the efficiency of DL methods in image classification tasks. The Inception V3 model presents a new convolutional network design that includes convolutional filters of different sizes in the single module to enable multi-scale processing. This design successfully captures features of all sizes and complexity, making it a crucial component of our research due to its capability to process varied images material and its impact on computing efficiency. EfficientNet B7 balances depth, width, and resolution in a balanced way using a novel scaling technique, resulting in exceptional efficiency and accuracy in image classification tasks. The architecture is specifically created to efficiently expand CNNs, showcasing the latest advancements in flexible and effective DL models. This makes it essential for assessing modern image processing methods.

Finally, using VGG 16 enhances the architectural simplicity of our study. The consistent architecture of VGG 16, which includes a series of convolutional layers that progressively become deeper and more intricate, serves as a foundation for comprehending the impact of depth and simplicity in network architecture on acquiring hierarchical feature representations. The model’s simple structure allows for the analysis of internal representations and the understandability of the acquired characteristics, which is crucial for the progress of explainable AI in image processing. Every model was carefully selected based on its unique contributions to the progress of CNNs and to provide a well-rounded representation of DL architectures. This selection highlights our dedication to examining the delicate equilibrium of model complexity, performance, interpretability, and computing efficiency. We want to enhance the current discussion on ethical responsibility and transparency in artificial intelligence by examining these models, emphasizing the importance of high-performance models that maintain the ideals of explainability and accountability in AI systems. Our study aims to enhance the possibilities in image processing using a complete approach, making sure that developments in AI are accessible, comprehensible, and morally responsible.

### 3.4. Experiments Protocol

We carefully created two experimental procedures to investigate and showcase the efficiency of sophisticated DL models in medical imaging, particularly for segmenting and classifying lung disorders in CXR images. The first technique utilizes the Attention U-Net model, known for its accuracy in segmenting multi-class CXRs by emphasizing important regions utilizing attention processes. The second approach utilizes the computational capabilities of ViTs for classification by including segmented CXRs from the Attention U-Net to improve the accuracy of illness detection. The methods, supported by a strong experimental setup and thorough assessment metrics, strive to expand AI’s diagnostic skills, providing new opportunities to enhance clinical outcomes in pulmonary illness detection.

In this section, we delve into the detailed methodologies employed in our study, outlining the two distinct experimental protocols that form the core of our investigation. These protocols have been meticulously crafted to assess the capabilities and performance of cutting-edge DL architectures in the context of medical image analysis, specifically focusing on the segmentation and classification of lung diseases in chest X-ray (CXR) images. Through the application of these experimental protocols, we aim to not only evaluate the effectiveness of these advanced models but also contribute to the ongoing efforts in enhancing diagnostic procedures and patient care in the medical field.

#### 3.4.1. Experimental Protocol 1: Segmentation Using Attention U-Net

Experimental Protocol 1 utilizes the Attention U-Net architecture to segment multiclass CXRs. The enhanced model uses attention processes to enhance the segmentation process, allowing for an accurate delineation of different lung illnesses. The technique includes an initial stage of data preparation in which CXR images are gathered, labeled with accurate information on various lung diseases, and processed to guarantee consistency in image quality and dimensions. The attention U-Net model is then trained on the selected dataset. The training process is carefully planned to improve the model’s performance by using stratified K-fold (k = 10) cross-validation and early stopping techniques. Performance measures like the Dice Coefficient and Jaccard Index are tracked to assess the accuracy of segmentation. The attention layers of the model concentrate on certain locations in the CXRs that are important for precise illness detection and segmentation. This protocol intends to test the performance of the attention U-Net in medical image segmentation and examine its potential to enhance diagnostic procedures by offering comprehensive and accurate visual representations of pulmonary problems.

#### 3.4.2. Experimental Protocol 2: Classification Using Vision Transformers

Experimental Protocol 2 focuses on categorizing lung disorders from CXR images using ViTs. It includes a pre-segmentation stage using Attention U-Net to emphasize lung areas. This method utilizes the worldwide contextual skills of ViTs like MobileViT, ViT Base, DEIT, and ViT Large to distinguish between lung diseases with improved accuracy. We used a stratified K-fold (k = 10) cross-validation procedure to ensure a thorough and balanced examination of diverse illness presentations in the dataset. Our protocol aims to establish new standards in the accuracy and reliability of lung disease diagnosis from CXRs by using a rigorous validation approach, ViTs, and Attention U-Net segmentation, moving towards improving clinical outcomes through the application of cutting-edge AI technologies.

### 3.5. Experimental Setup and Loss Function

All models were trained using the GPU cluster at Idaho State University (ISU). The PyTorch library facilitated the development of the AI system. Common hyperparameters in models include the optimizer Adam. The learning rate is 0.0001. The loss function used is categorical cross-entropy, with a batch size of 64, classification activation function is softmax, and the models are trained for 50 epochs with early stopping.

The Cross-entropy (CE) loss function is fundamental in training DL models for classification problems. The metric functions as a performance indicator that assesses how well the model’s projected probability matches the actual labels. The cross-entropy loss evaluates the expected probability ascribed to the correct class label for a certain prediction. When the forecast is accurate and confident, the loss is minimal; however, if the prediction is confident but inaccurate, the loss is significant. CE loss is well suited for training classification models since it penalizes inaccurate and confident predictions efficiently.

The function is calculated by obtaining the negative logarithm of the anticipated probability for the correct class. Binary classification tasks require the computation of separate terms for the positive and negative classes, which are then combined to obtain the final loss for each data point. Minimizing the cross-entropy loss during training enables the model to push its predictions closer to 0 or 1, indicating more confidence for the negative or positive class, respectively. The objective of the optimization process is to modify the model parameters to minimize the cross-entropy loss on the whole training dataset, resulting in optimal generalization performance on new data.

### 3.6. Evaluation Metrics

We used several performance assessment criteria that relied on True Positive (TP), True Negative (TN), False Positive (FP), and False Negative (FN) data. The evaluation metrics used were accuracy (*ɳ*), recall (*Ɍ*), precision (*Ƥ*), and the F1-score (*Ƒ*). We computed the Dice (Đ(ƴ,ƶ)*)* [51] and Jaccard (I(ƴ,ƶ)*)* [52] coefficients to assess segmentation similarity, with *ƶ* representing the required items and *ƴ* representing the discovered items. We evaluated the diagnostic potential by analyzing the ROC curve and calculating the AUC.

(1) $$ɳ=\frac{TP+TN}{TP+FP+FN+TN}$$

(2) $$Ɍ=\frac{TP}{TP+FN}$$

(3) $$Ƥ=\frac{TP}{TP+FN}$$

(4) $$Ƒ=2*\frac{Ƥ*Ɍ}{Ƥ+Ɍ}$$

(5) $$Đ(ƴ,ƶ)=2\frac{\left|ƴ\right||ƶ|}{\left|ƴ\right|+|ƶ|}$$

(6) $$I(ƴ,ƶ)=\frac{Đ(ƴ,ƶ)}{2-Đ(ƴ,ƶ)}$$

## 4. Results

This section of the research rigorously evaluates the use of robust DL models for diagnosing and segmenting lung diseases using chest X-rays. It showcases the enhanced accuracy of U-Net with attention mechanisms by illustrating their efficacy with high Dice Coefficients and Jaccard Indexes. The section provides an extensive examination of model performances in multi-class classification of COVID-19, highlighting the accuracy of Transformer-based classifiers and Attention U-Net. The findings highlight the importance of using attention mechanisms with Transformer technology in enhancing medical imaging analysis via quantitative measures and visual comparisons. This advancement aims to improve the accuracy, reliability, and interpretability of diagnostic procedures in healthcare.

### 4.1. Lung Segmentation Results

The research analyzes how U-Net with attention mechanisms function in segmenting lung illnesses from chest X-rays, demonstrating their effectiveness in medical image processing. The U-Net with attention mechanisms is a model designed to enhance the precision of segmentation tasks. The model has shown exceptional performance, with a Dice Coefficient of 98.54% and a Jaccard Index of 97.12%. The data suggests that the model can accurately delineate affected lung sections, highlighting its improved ability to capture the intricate limits and variations within the pulmonary systems.

The U-Net with attention mechanisms has high Dice Coefficient and Jaccard Index, highlighting its effectiveness in precisely segmenting lung illnesses. This validates its potential as a strong segmentation tool. The model’s performance demonstrates the benefits of including attention mechanisms into segmentation networks and signifies advancements in using DL for medical image segmentation. The U-Net with attention mechanisms enhances the early detection and precise classification of lung illnesses by providing more accurate and detailed segmentations, resulting in improved clinical outcomes.

**Figure 7:**
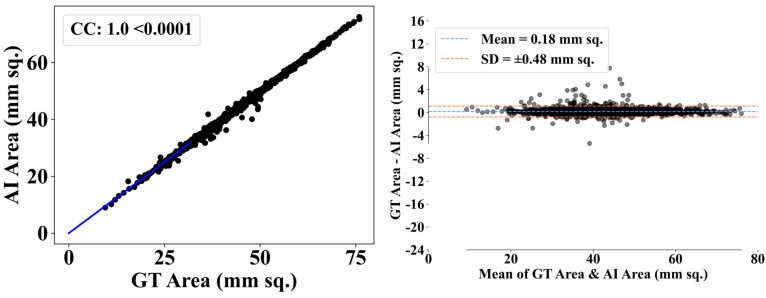
Comparative analysis of ground truth (GT) versus algorithmic measurement area estim −tions with correlation coefficient (CC) and Bland–Altman plot [53] assessment.

Figure 7 presents the AI’s area estimates compared to the benchmark ground truth (GT) data. The scatter figure on the left demonstrates a robust positive linear connection between the AI and GT area measurements, as shown by a correlation coefficient (CC) of 1.0. This signifies a flawless linear relationship. The Bland–Altman figure on the right shows the amount of agreement between the two approaches, with most data points clustering around a mean difference near to zero, indicating low bias in the AI’s estimates compared to GT. The tiny standard deviation indicates a narrow distribution of variances around the mean, showing consistent performance by the AI across various area sizes. The plots provide a thorough assessment of the AI’s accuracy in determining the region, confirming its high reliability and close agreement with the ground reality.

**Figure 8:**
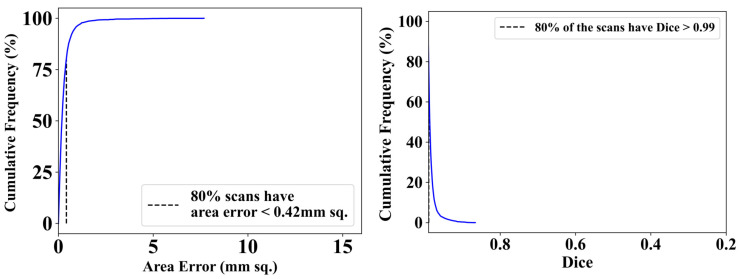
Cumulative distribution of area errors and Dice similarity coefficients for algorithmic scans.

Figure 8 presents two graphs that measure the scanning efficiency of an algorithm. The graph on the left displays the cumulative percentage of scans in relation to area error. It reveals that 80% of scans have an area error below 0.42 mm sq., suggesting that mistakes are mostly modest, since the curve sharply increases approaching this threshold. This indicates a high degree of precision within the lower margin of error. The graph on the right shows the cumulative distribution of Dice coefficients, which is a statistical measure of similarity between the algorithm’s output and a standard reference. Over 80% of scans have a Dice coefficient higher than 0.99, indicating a strong agreement with the reference. Occurrences with lower Dice coefficients are rare. These curves provide essential data for assessing the algorithm’s reliability and accuracy, especially in medical imaging analysis applications where these metrics are critical.

**Figure 9:**
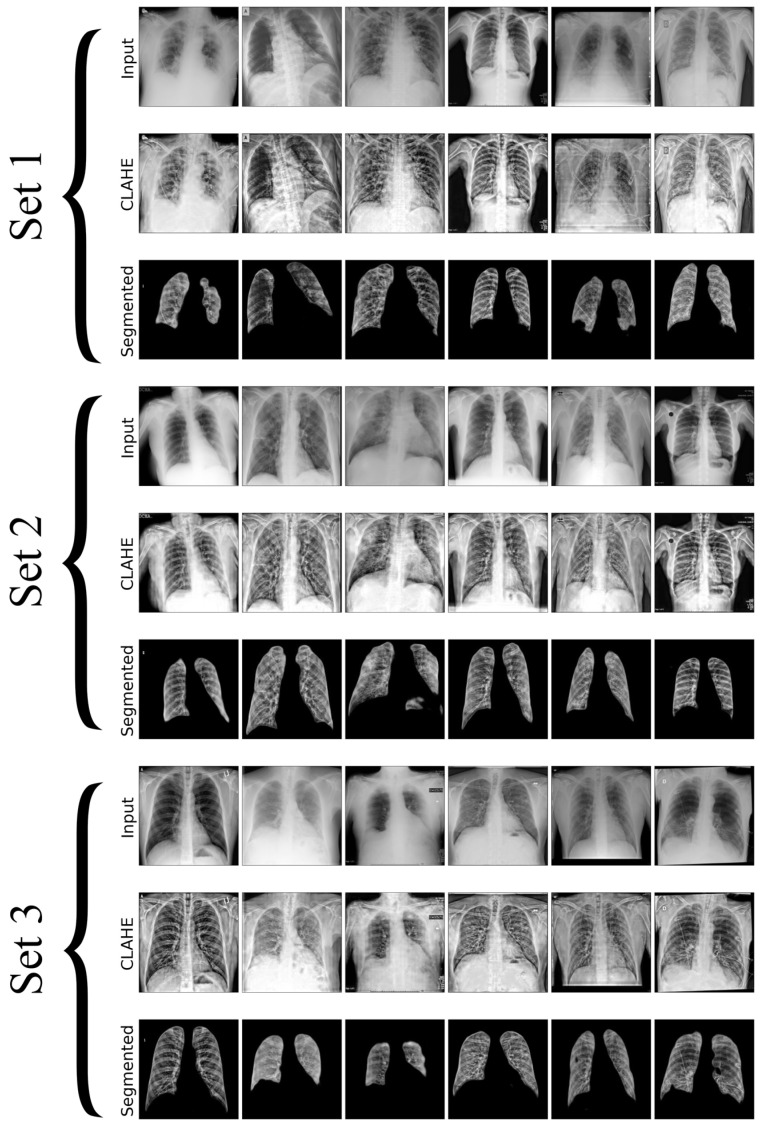
Side-by-side comparison of a chest X-ray before CLAHE enhancement, after CLAHE enhancement, and segmented images.

Figure 9 presents a visual matrix with nine rows grouped into three sets, each including three rows that demonstrate several steps of image processing for medical imaging analysis. The first row of each group displays the original X-ray pictures, serving as a reference for comparison. The second row displays the improved pictures achieved by using CLAHE, a method that enhances image contrast to facilitate the identification of important elements. The third row in each group shows the segmented pictures processed using an attention U-Net architecture. The convolutional network utilizes attention techniques to enhance the accuracy and precision of the segmentation process by focusing on important portions of the picture. The figure demonstrates the process of transforming the original input into a clearer version and then into a segmented output, showcasing the effectiveness of the techniques used to extract and emphasize important anatomical components for medical examination.

### 4.2. Multi-Class Classification of Lung Diseases

Multi-class classification is critical in analyzing CXRs to identify lung disorders, including normal cases, bacterial pneumonia, viral pneumonia, COVID-19 cases and tuberculosis. These labels were marked by radiologists. This method enables a comprehensive evaluation of various pulmonary conditions, enhancing diagnostic accuracy and improving patient outcomes by providing a more detailed and nuanced understanding of each condition [54,55,56,57,58,59,60,61]. Our study evaluated Transformer-based deep learning classifiers, measuring their performance using accuracy, F1-score, recall, and precision. The results demonstrated the classifiers’ robust ability to distinguish between different lung conditions, highlighting their potential applicability in real-world clinical scenarios.

Table 1 presents a detailed comparison showing that the field of medical image classification, particularly in diagnosing lung disorders from chest X-rays, is undergoing a notable transformation by using ViT models and hybrid methods such as MobileViT. The metrics provided show significant differences in performance amongst various models. MobileViT stands out with an accuracy of 98.52%, along with precision, recall, and F1-score, each close to 98.5%. The consistency in key performance parameters showcases MobileViT’s effectiveness in precise illness diagnosis with few errors, establishing it as a top option for clinical diagnostic purposes.

The ViT Large model’s performance is impressive, achieving an accuracy of 98.12% and nearly matching scores in precision, recall, and F1, as shown in Table 1. The findings highlight the benefits of using larger and more advanced Transformer models for in-depth analysis in medical imaging, which allows for accurate diagnosis. The addition of Transformer-based models such as MobileViT, ViT Base, and ViT Large highlights a transition from conventional CNN approaches to ones that can efficiently use global contextual analysis for classification. ViTs excel at activities that need detailed comprehension and classification of intricate visual patterns, such those seen in medical diagnostics, because of their capacity to examine pictures comprehensively.

The information presented in Table 1 provides a convincing account of the capabilities and benefits of Transformer models in the field of medical imaging. The text emphasizes a change in approach towards using models that combine global and local feature analysis, beyond the constraints of conventional CNNs.

## 5. Comparative Analysis

In medical image analysis, the selection of model architecture significantly influences diagnostic accuracy and result reliability. Comparing CNNs with ViTs reveals insights into the changing field of DL in medical imaging. We can determine the greater ability of ViT models to capture global contextual information compared to typical CNNs by analyzing important performance parameters including accuracy, F1-score, recall, and precision. This investigation highlights the benefits of switching to Transformer-based designs to improve diagnostic results in medical imaging activities.

### 5.1. Classification Using Raw Input Data

**Figure 10:**
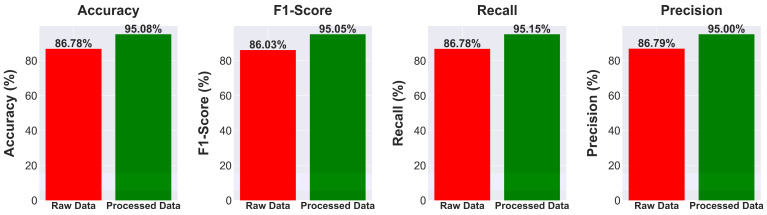
Comparison between raw data classification and processed data classification.

The quantitative analysis comparing model performances on data processed using CLAHE and segmentation (Table 1) to models trained on raw input data (Table 2), revealed the benefits of both approaches. Using CLAHE and segmentation, MobileViT achieves an accuracy of 98.52%, a notable increase compared to its performance on raw data, which drops to 90.31%. The ViT Large model shows a significant decrease in accuracy, dropping from 98.12% with CLAHE and segmentation to 88.40% with raw input. Transitioning from data enhanced with CLAHE and segmentation to raw data in all models resulted in a noticeable decrease in key performance metrics such as accuracy, F1-score, recall, and precision. Utilizing CLAHE and segmentation approaches, as seen in Figure 10, greatly improves the model’s performance. An average accuracy gain of 8.3%, a 9% increase in the F1-score, an 8.37% boost in recall, and an 8.21% rise in precision were seen across various models. The findings highlight the efficacy of these strategies in improving model outcomes.

The significant numerical evidence emphasizes the crucial role of using CLAHE and segmentation to improve data quality for machine learning models. This dual method overcomes the constraints of raw data and enhances the model’s learning capacity by providing precise, contrast-enhanced pictures highlighting certain aspects of interest. The significant improvement in model accuracy and performance measures after using CLAHE and segmentation demonstrates the effectiveness of these strategies in improving data inputs. Enhancing data quality via CLAHE and segmentation significantly enhances the effectiveness of DL models, particularly in situations requiring high precision and accuracy. The research strongly advocates for using CLAHE and segmentation as crucial elements in the data preparation process to ensure that models are trained on well-prepared data, leading to enhance learning efficacy and outcome reliability.

### 5.2. U-Net vs. Attention U-Net

**Figure 11:**
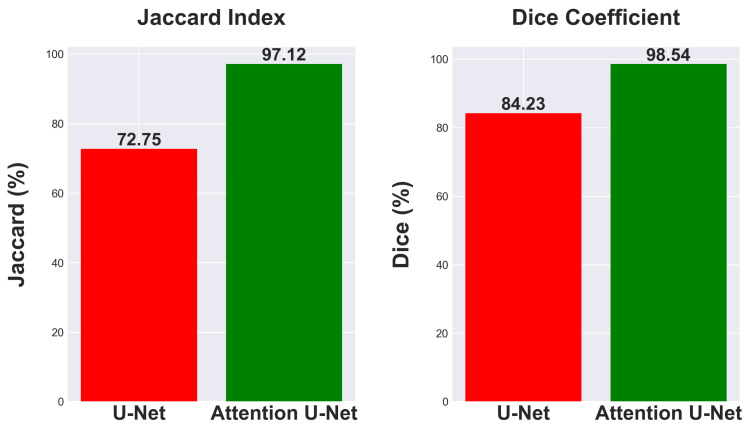
Jaccard and Dice comparison between U-Net and Attention U-Net.

This research investigates the effectiveness of using Attention U-Net for segmentation tasks and compares its performance to the traditional U-Net architecture. Comparing segmentation metrics with and without attention mechanisms in neural network designs is crucial for demonstrating advances. The traditional U-Net model, known for its efficacy in segmentation tasks, achieved a Jaccard Index of 72.75% and a Dice Coefficient of 84.23% as seen in Figure 11.

The Attention U-Net, which enhances the segmentation process by emphasizing important characteristics using attention gates, showed a significant increase in performance. The model attained a Dice Coefficient of 98.54% and a Jaccard Index of 97.12%, as seen in Figure 11. The findings emphasize the benefits of integrating attention processes into neural network architectures for segmentation, showcasing significant improvements in accuracy and reliability. The improved Dice Coefficient shows better agreement between the predicted segmentation and the ground truth, while the higher Jaccard Index indicates a more accurate match in the segmentation areas, highlighting the model’s ability to differentiate effectively between the region of interest and the background.

The attention mechanism of the Attention U-Net improves segmentation metrics by focusing on the most relevant features in an image. It demonstrates a 24.37% enhancement in Jaccard index and a 14.31% rise in Dice coefficient, showcasing its better performance in image segmentation tasks. It is a promising method for intricate segmentation tasks where precision is paramount.

### 5.3. Convolutional Neural Networks vs. Vision Transformers

When comparing CNN and ViT models for medical image classification, significant performance disparities are seen, especially in the area of diagnosing lung diseases using CXRs. Table 3 summarizes the results of several models based on important measures such as accuracy, F1-score, recall, and precision.

Starting with CNNs, ResNet 50, a renowned model known for its depth and capacity to capture complex information via convolutional layers, achieves an accuracy of 66.30%. The performance metrics, such as an F1-score of 65.53%, recall of 66.88%, and precision of 66.74%, indicate that the model excels in image classification tasks but may face limitations in medical image analysis due to the CNN’s tendency to prioritize local features over global context. Transformer variants such as MobileViT and ViT Large achieve much greater accuracies of 98.52% and 98.12%, respectively. Both models have a strong correlation in precision, recall, and F1-score, demonstrating good accuracy and a balanced capacity to reduce both false positives and false negatives. The improvement in performance is due to the Transformer’s capability to process global contextual information, which is beneficial in medical imaging tasks that require distinguishing between categories based on minor, global distinctions that may not be caught by the local receptive fields of CNNs.

Additionally, VGG 16 and EfficientNet B7 CNN models outperform ResNet 50, with VGG 16 obtaining 98.34% accuracy and EfficientNet B7 reaching 89.80%. The variability in CNN performance suggests that enhancements and advancements and iterations on CNN structures result in improvements, while they fall short of the most recent Transformer-based models in this particular use case. VGG 16, which performs similarly to Transformer models, indicates that deep CNNs with architectural improvements may still excel in challenging applications such as medical image categorization.

**Figure 12:**
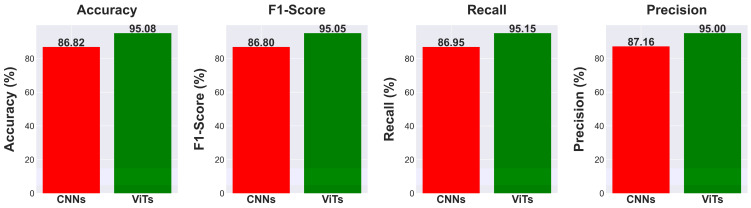
Comparison between CNNs and ViTs.

Figure 12 shows a clear difference in performance indicators between CNN and ViT models. ViT models often achieve an accuracy of 95.08%, surpassing CNNs that have an average accuracy of 86.82%. ViTs outperform CNNs in F1-Score, with an average of 95.05% compared to CNN’s 86.80%. ViTs have an average recall metric of 95.15%, whereas CNNs have an average of 86.95%. The precision metric for ViTs is 95.00%, above the CNN average of 87.16%. ViT models surpass CNNs by about 8.26% in accuracy, 8.25% in F1-Score, 8.20% in recall, and 7.84% in precision on average, as shown by the statistics. The substantial performance difference indicates that ViT models may be more appropriate for tasks where accuracy and precision are crucial. The evident superiority of ViTs compared to CNNs in all evaluated criteria suggests a possible inclination towards Transformer-based structures in domains that heavily depend on image classification and analysis, like medical imaging, where improving accuracy and reliability could significantly influence patient outcomes.

## 6. Performance Evaluation

**Figure 13:**
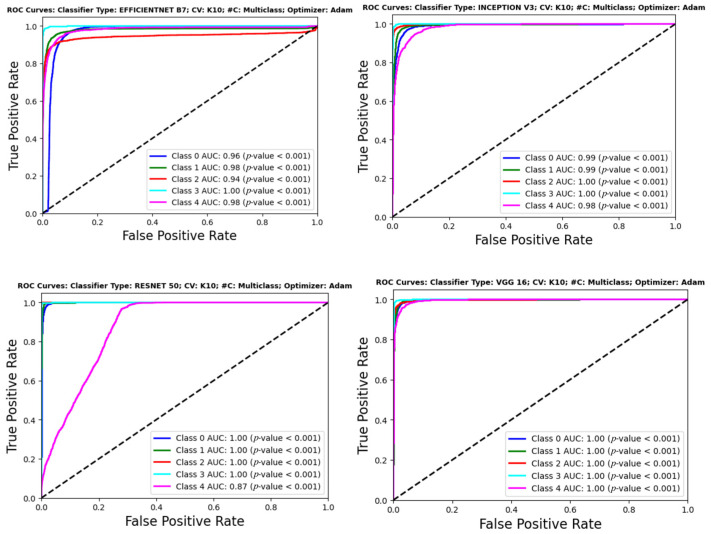
ROC curves for CNN models.

It is crucial to thoroughly test the effectiveness of DL models in categorizing lung disorders using CXRs to guarantee the credibility and reliability of the models. Figure 13 displays the ROC curves of several CNN classifiers, such as EfficientNet B7, Inception V3, ResNet 50, and VGG 16. These classifiers exhibit excellent discriminative performance, with AUC values often nearing the optimal score of 1.00 for many classes. The high AUC values, constantly accompanied by statistically significant *p*-values (*p* < 0.001), confirm the model’s outstanding capability to differentiate between normal and abnormal results on chest X-rays.

**Figure 14:**
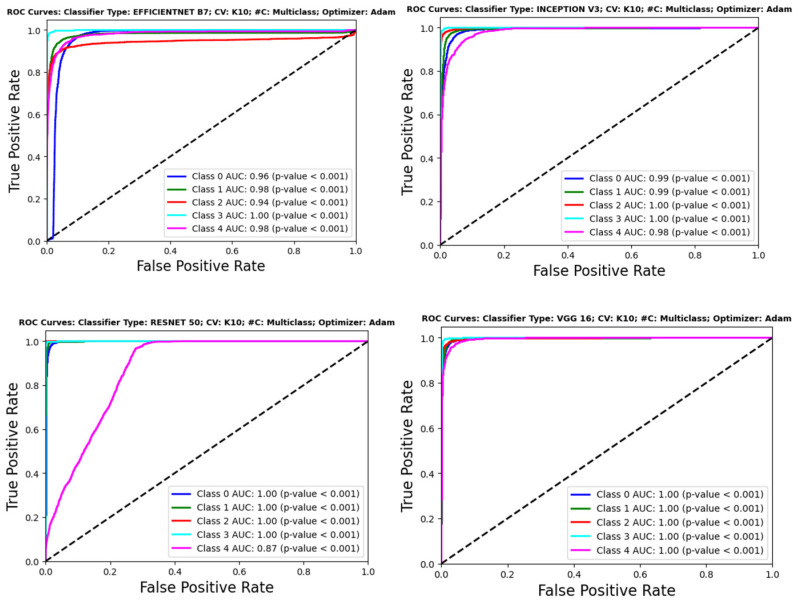
ROC curves for Transformer models.

Figure 14 showcases the performance of transformer-based models such as ViT Base, ViT Large, and the Data-efficient Image Transformer (DeiT). The ViT Large and ViT Base models demonstrate remarkable accuracy across all classes, with the ViT Large model almost obtaining flawless AUC values. The DeiT’s performance is outstanding as well, with AUC values over 0.97 for all classes, showcasing its good classification power while not reaching the highest values. This is particularly noteworthy due to its data-efficient architecture.

Upon careful examination of ResNet 50’s ROC curve in Figure 13, there is a subtle decrease in performance seen for the tuberculosis class (Class 4), indicating potential challenges in capturing the unique features of this illness. This decline in performance highlights the need to improve the model or enhance the dataset for illnesses with minor radiographic characteristics. The MobileViT model, shown in Figure 14, demonstrates the effectiveness of transformer topologies in processing medical imaging data, addressing global dependencies, and achieving high AUC values for different classes, as compared to VGG 16, ViT Base, and ViT Large.

The slight differences in performance shown across the models for various classes emphasize the importance of selecting a model that suits the precise details and requirements of the diagnostic job. In circumstances when illnesses like TB have less obvious radiographic signs, advanced feature extraction and learning algorithms are needed to enhance detection and classification rates. ROC curves offer a quantitative method to assess the effectiveness of models in clinical settings, ensuring that the selected model meets the specific task requirements, such as identifying diseases like tuberculosis that are clinically significant despite potentially lower performance. The results have two primary ramifications. They demonstrate the advanced AI capabilities in medical diagnostics with tools that can improve the doctor’s expertise, with high precision and reliability. Secondly, they stress the need for continuous research and development in this sector to improve these models, ensuring they are both successful and equitable in diagnosing different diseases and patient groups. This performance assessment indicates that DL models are not only theoretical exercises but are also sophisticated tools suited for use in clinical settings. The models have high AUC values across most classes, suggesting the possibility of enhancing the diagnostic process. This improvement might lead to earlier detection and better patient outcomes in managing pulmonary illnesses.

## 7. Statistical and Reliability Analysis

Statistical tests are important components for evaluating the reliability and stability of the AI systems [38,58,62,63,64]. Our study extensively analyzes the effectiveness of four ViTs using a rigorous statistical approach to evaluate their performance. Our methodology comprises two primary statistical tests: Tukey’s Honestly Significant Difference test [58], and McNemar test. Every test has a unique function in assessing both statistical significance and practical relevance across the models. The objective is to create a ranking system for model performance using factual data, shifting away from anecdotal evidence to depend on statistical precision [65].

Tukey’s Honestly Significant Difference (HSD) test [66] was used after conducting an ANOVA to enable thorough pairwise comparisons amongst machine learning models including DeiT, ViT Base, ViT Large, and MobileViT. This post hoc test was specifically created to precisely detect noteworthy performance variations across groups, while also considering the likelihood of Type I errors in numerous comparisons. The HSD test is effective in offering a precise and statistically strong method for comparing several groups without increasing the likelihood of false positives.

The HSD test findings in Table 4 provide important insights into the comparative performances of the different models being studied. The DeiT model shows statistically significant performance differences compared to other models like ViT Base, ViT Large, and MobileViT, with *p*-values below 0.001. The statistical significance is supported by the mean differences found, indicating the higher performance of DeiT. DeiT performs better than ViT Base with a significant mean difference of 0.0837, and this difference is much more evident when compared to ViT Large and MobileViT. The significant mean differences of 0.0921 and 0.0677 emphasize DeiT’s superior efficiency and competence in the tested settings. Moreover, MobileViT exhibits statistically significant distinctions when contrasted with the other models, highlighting the diverse performance levels of these advanced technologies. However, as shown by a *p*-value of 0.3386, statistical analysis did not show a significant difference between the performance of ViT Base and ViT Large.

Tukey’s HSD test study shows that the DeiT model outperforms ViT Base, ViT Large, and MobileViT. It also reveals that there is no significant performance difference between ViT Base and ViT Large, with a *p*-adj value of 0.3386. The absence of statistical significance might be due to the identical structural design of ViT Base and ViT Large, indicating that despite differences in size or capability, the fundamental architectural elements of both models result in equivalent efficacy. This discovery highlights the significance of architectural subtleties in influencing model performance and stresses that simple enhancements in model size or complexity do not necessarily result in substantial performance improvements. The precise findings from the HSD test provide for a more sophisticated understanding of model performance, which may help direct future research efforts towards improving architectural designs and concentrating on innovations that really boost model effectiveness. This work helps in choosing the most efficient models for certain tasks and contributes to the advancement of the machine learning industry by emphasizing the crucial role of architecture in model effectiveness.

The McNemar test [67], which we used in our research, is a particular non-parametric statistical approach designed for assessing paired nominal data. This test is very skilled at evaluating the consistency of predicted accuracies between two DL models when used on the same dataset. The main benefit of this method is its capability to assess whether variations in predicted accuracy are statistically meaningful, offering a detailed view on comparing models, which is particularly important for binary classification assignments.

The McNemar test findings shown in Table 5 show statistically significant differences in performance for all pairs of models (DeiT, ViT Base, ViT Large, and MobileViT), with *p*-values below 0.001. The results emphasize both the statistical significance of the performance differences found and their practical importance in selecting and implementing models in real-world scenarios. The consistently low *p*-values indicate significant and persistent variations in predicting accuracy, emphasizing the need of selecting models carefully using empirical data. The McNemar test offers a statistical basis for distinguishing between model performances, aiding researchers and practitioners in optimizing predictive accuracy and application success.

## 8. Explainability

The drive towards explainable AI, particularly in critical fields like medical diagnostics, aims to make complex models like CNNs and Transformers transparent and trustworthy. This is crucial for understanding model decisions, improving performance, and ensuring reliability in high-stakes environments. Techniques like LRP and Gradient-weighted Class Activation Mapping (Grad-CAM++) are essential in achieving this by providing insights into how and why models make certain predictions. These methods enhance trust, facilitate debugging, and highlight areas for model refinement, particularly in healthcare applications where accuracy and clarity are paramount.

### 8.1. Theory of LPR and Grad-CAM++

This study highlights the significance of explainability in ensuring that our DL models prioritize medically important information, such as lesions indicating lung diseases, above irrelevant image characteristics. We use LRP for Transformer models and Grad-CAM++ for CNN and hybrid models for this purpose. LRP provides insight into how Transformers make choices by highlighting key qualities that impact the result, which are ideal for their complex structure and self-attention mechanisms. Grad-CAM++ generates visual explanations for CNNs and hybrid models by pinpointing the important regions in images that influence the model’s predictions. We ensure the precision and lucidity of our analysis by using a dual procedure tailored to the unique characteristics of each model type. Our study aims to enhance the transparency and clinical significance of artificial intelligence in medical diagnostics by concentrating on key pathological features for detecting lung illnesses. This would boost confidence and implementation in clinical environments.

In this study, we employ Grad-CAM++ on Convolutional Neural Networks (CNNs) and hybrid models such as ResNet 50, VGG 16, EfficientNet B7, Inception V3, and MobileViT. The reason for using Grad-CAM is its design tailored to models with convolutional layers. By using Grad-CAM++, which enhances the weight calculation process using higher-order gradients, we can conduct a more comprehensive analysis of each pixel’s impact on the target class, thereby improving the interpretability of CNNs and hybrid models. LRP is preferred over Grad-CAM++ for Pure ViTs such as ViT Base, ViT Large, and DeiT to improve model interpretability due to their distinctive design. ViTs process images via self-attention processes, considering them as sequences of patches without spatial feature maps, unlike CNNs, which Grad-CAM++ was developed for. LRP is crucial for clarifying ViT’s internal decision-making process by revealing how features are prioritized, particularly in challenging tasks such as lung disease categorization. This decision guarantees a precise understanding of ViT’s results by matching their architectural intricacies and offering valuable insights into their operating mechanisms.

#### 8.1.1. Layer-Wise Relevance Propagation

LRP is a technique used to analyze neural networks, particularly beneficial for comprehending the decision-making mechanism of DL models. It operates by using the concept of relevance backpropagation. The process starts with the network’s ultimate output selection, often a classification score. The primary idea is to attribute the output choice to the input layer by propagating the output score across the network layers.

- Propagation Rule: LRP employs precise criteria, which are often tailored to individual layers, to systematically transfer importance from upper levels to lower layers. These criteria, based on the conservation of relevance, guarantee that the overall relevance in a layer is identical to the relevance assigned to the following layer.
- Pixel-Level Attribution: Relevance is back-propagated to the input layer, assigning a relevance score to each pixel in the input image. This score indicates the impact of each pixel on the final decision.
- Visualization: The LRP result is often shown as a heatmap superimposed on top of the original input image. The heatmap illustrates the pixels and places with the most significant influence on the model’s decision-making process.

(7) $$R_{i}=\sum_{j}\frac{a_{i}w_{ij}}{\sum_{i^{\prime }}a_{i^{\prime }}w_{i^{\prime }j}}R_{j}$$

The core equation of LRP is a rigorous mathematical method used to systematically assign the output decision of a neural network to its input features. $R_{I}$ in this equation denotes the importance assigned to the *i*-th neuron in a certain layer, signifying its role in the network’s final decision. $R_{j}$ signifies the relevance attributed to the *j*-th neuron in the subsequent layer, which is the portion of the output decision accounted for by that neuron. LRP maintains relevance conservation at each layer by iteratively backpropagating from the output layer to the input across the network, where $a_{i}$ represents the activation of the neuron and $w_{ij}$ represents the weight of the connection. The meticulous process of backpropagation continues until the relevance scores reaches the input layer, accurately identifying the importance of individual pixels to the model’s conclusion. Within deep neural networks, this method is very beneficial since it clarifies the network’s conclusions at a detailed, pixel level, significantly improving model transparency and interpretability.

#### 8.1.2. Gradient-Weighted Class Activation Mapping

Grad-CAM++ enhances the interpretability of CNNs by providing a sophisticated method for creating high-resolution class activation maps, overcoming the constraints of its predecessor, Grad-CAM. A sophisticated method is shown for determining the significance weights of feature maps and producing precise class activation maps that emphasize the crucial areas influencing a model’s choice. The primary components of Grad-CAM++ are summarized in two fundamental formulas:

- Weight Calculation for Each Feature Map ($\alpha_{k}^{c}$): Grad-CAM++ enhances the weight calculation algorithm by using higher-order gradients, allowing for a more detailed evaluation of the contribution of each pixel to the target class. This is a substantial improvement compared to Grad-CAM’s average gradient method. The weight ($\alpha_{k}^{c}$) for each feature map (*k*) related to a target class (*c*) now considers the intricate, non-linear relationships between the feature map activations (*A*) and the class output score ($y^{c}$). The updated algorithm for determining these weights includes partial derivatives up to higher orders to accurately capture how each feature map activation impacts the class prediction.
- Localization Map Generation ($L_{\mathrm{Grad}-\mathrm{CAM}++}^{c}$): Grad-CAM++ calculates the localization map ($L_{\mathrm{Grad}-\mathrm{CAM}++}^{c}$) by combining the feature map activations with refined weights ($\alpha_{k}^{c}$) and using a Rectified Linear Unit (ReLU) function. This method is similar to Grad-CAM but utilizes more discriminatively determined weights, resulting in a graphic that more clearly highlights the significant regions for the specific class. The localization map formula effectively integrates the contributions of all feature maps, highlighting aspects that positively influence the prediction of the target class.

Mathematically, the weight computation in Grad-CAM++ involves using the global average pooling of gradients and adding factors that account for higher-order interactions between feature map activations and class scores. This technique excels at analyzing the model’s focus in more detail than Grad-CAM, offering deeper insights into the model’s behavior with enhanced granularity.

(8) $$\alpha_{k}^{c}=\frac{1}{Z}\sum_{i}\sum_{j}\left(\frac{\partial^{2}y^{c}}{\partial A_{ij}^{k2}}\right)\frac{\partial y^{c}}{\partial A_{ij}^{k}}+\left(\frac{\partial^{3}y^{c}}{\partial A_{ij}^{k3}}\right)$$

(9) $$L_{\mathrm{Grad}-\mathrm{CAM}++}^{c}=\mathrm{ReLU}\left(\sum_{k}\alpha_{k}^{c}\cdot A^{k}\right)$$

Grad-CAM++ enhances the process for determining significance weights and creating localization maps, allowing for a more thorough and exact depiction of the factors influencing CNNs output. Grad-CAM++ is very helpful for jobs that need thorough interpretability, such as fine-grained categorization, detecting multiple occurrences in an image, and comprehending subtle features used by a model for its predictions.

### 8.2. Interpretation of Explainability

Explainability is essential in DL, especially in medical imaging, for understanding model choices, building clinician confidence, and revealing the model’s emphasis regions in diagnosis. The procedure incorporates methods that reveal the underlying decision-making of intricate models, emphasizing the characteristics or areas in an image that have the largest impact on the model’s forecast. Understanding the reasoning behind a model’s diagnosis is crucial in healthcare applications, as it aids in treatment planning and pinpointing areas for model enhancement.

**Figure 15:**
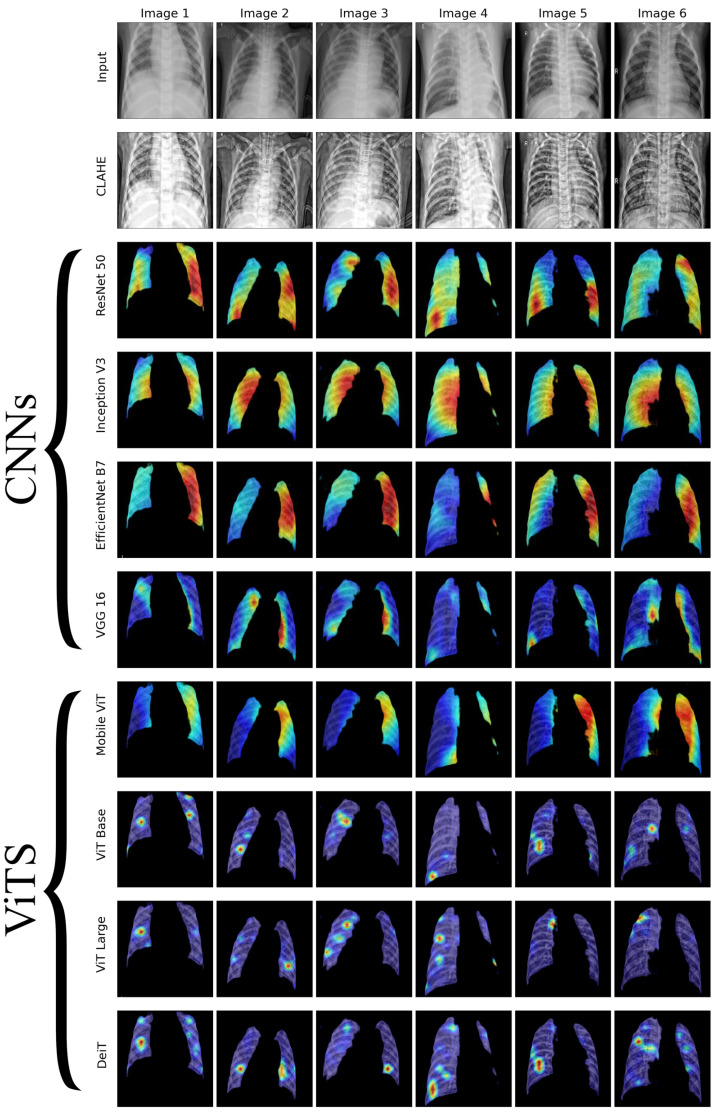
Heatmaps for bacterial classes.

**Figure 16:**
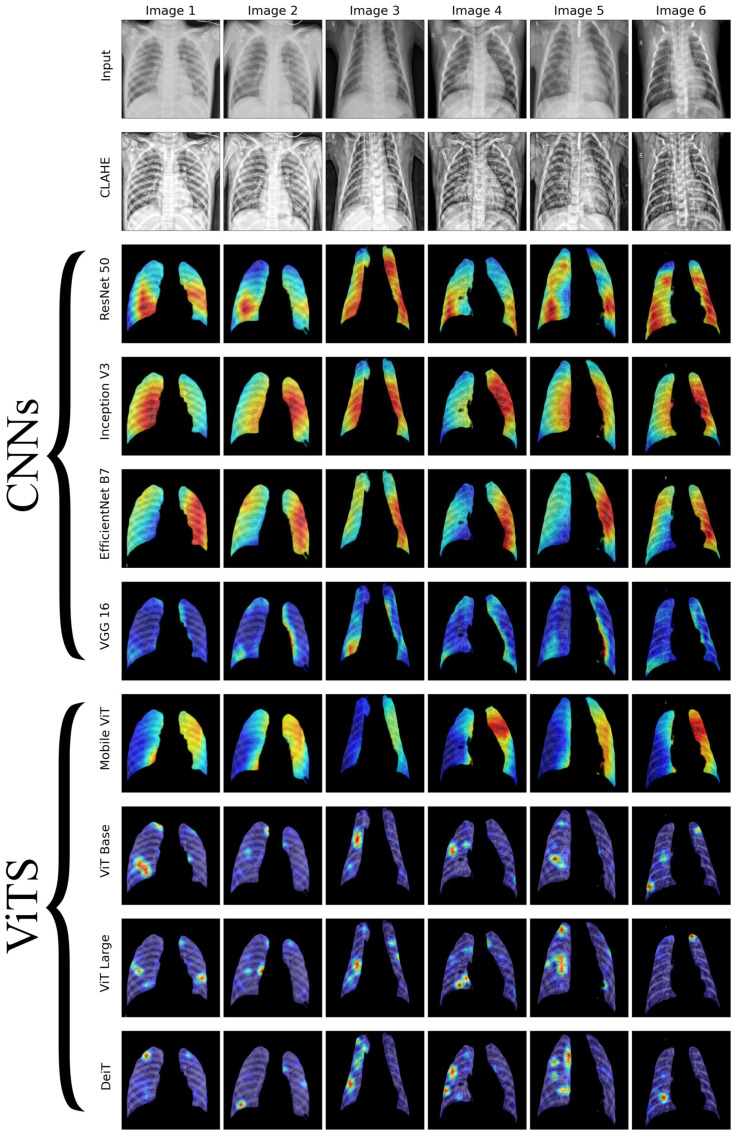
Heatmaps for bacterial classes.

**Figure 17:**
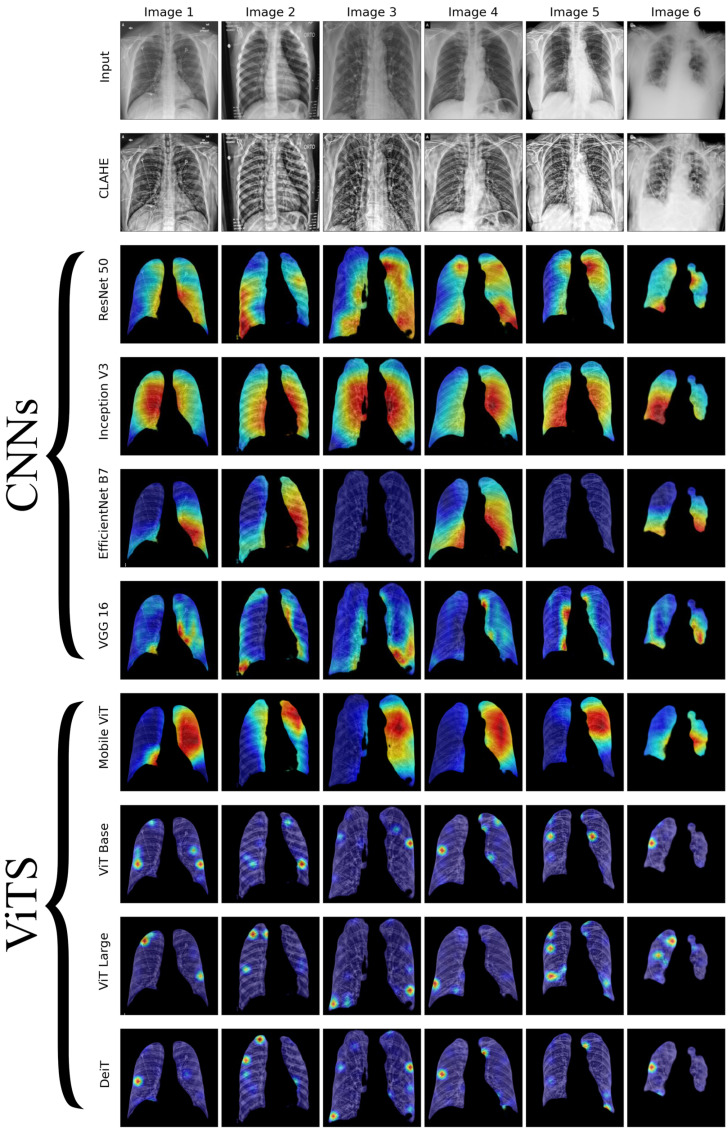
Heatmaps for COVID-19 classes.

**Figure 18:**
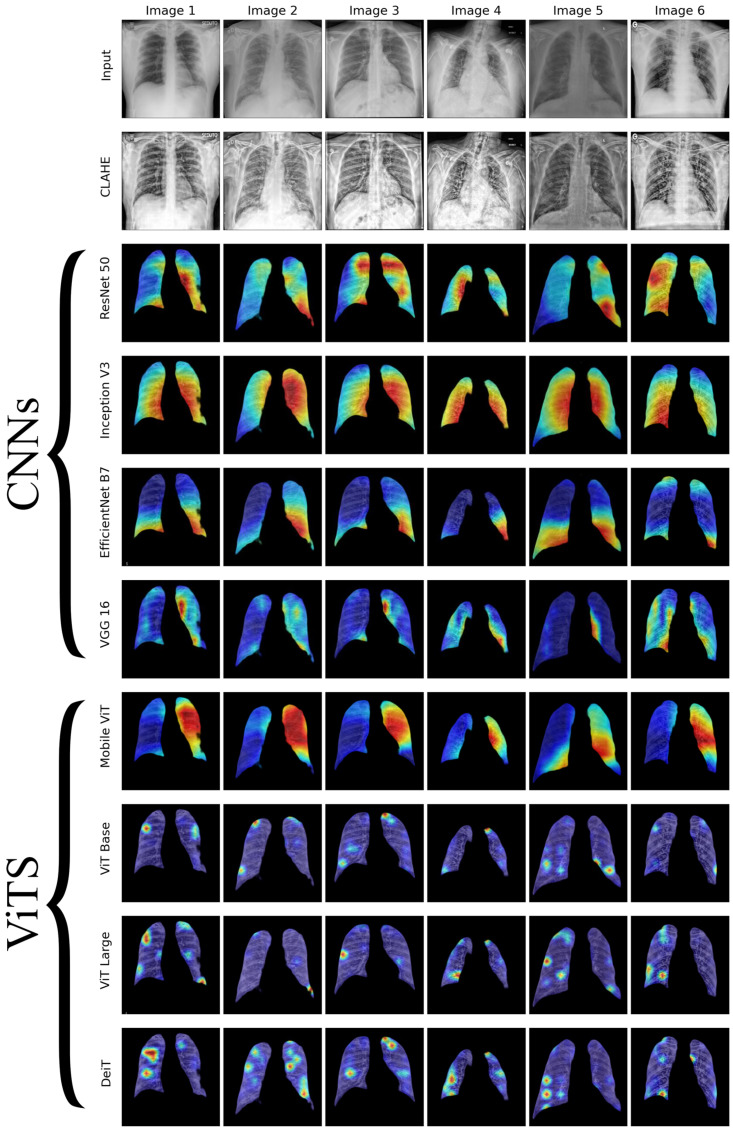
Heatmaps for COVID-19 classes.

**Figure 19:**
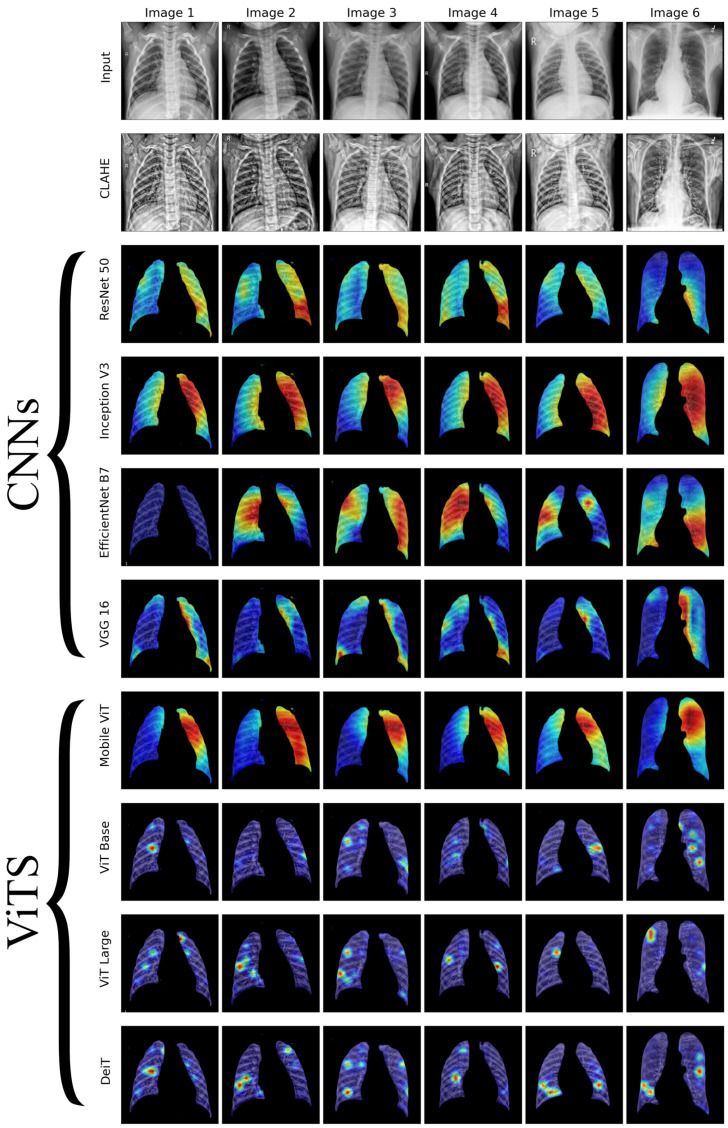
Heatmaps for normal classes.

**Figure 20:**
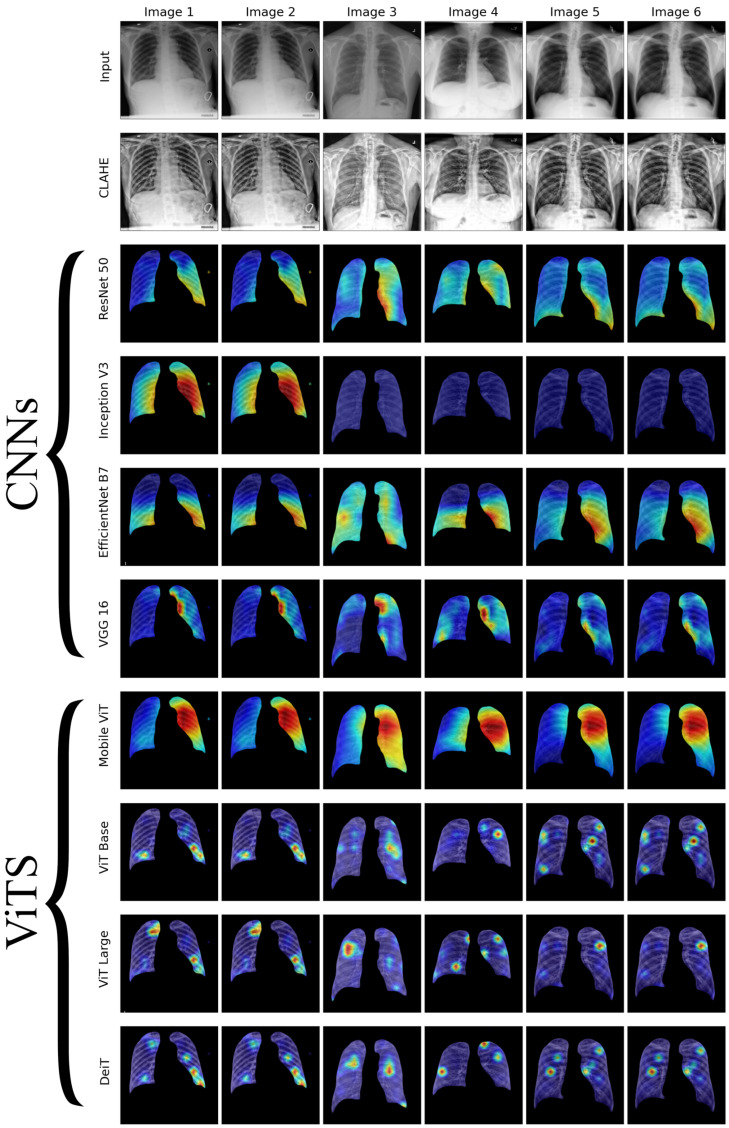
Heatmaps for normal classes.

**Figure 21:**
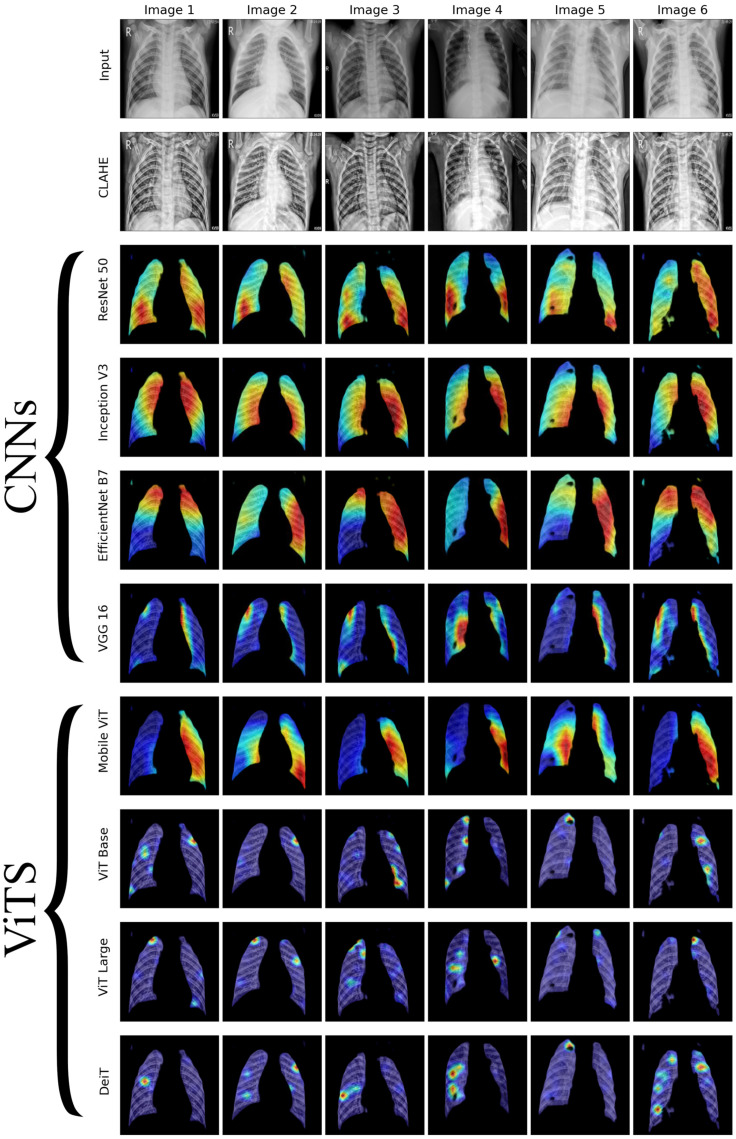
Heatmaps for viral classes.

**Figure 22:**
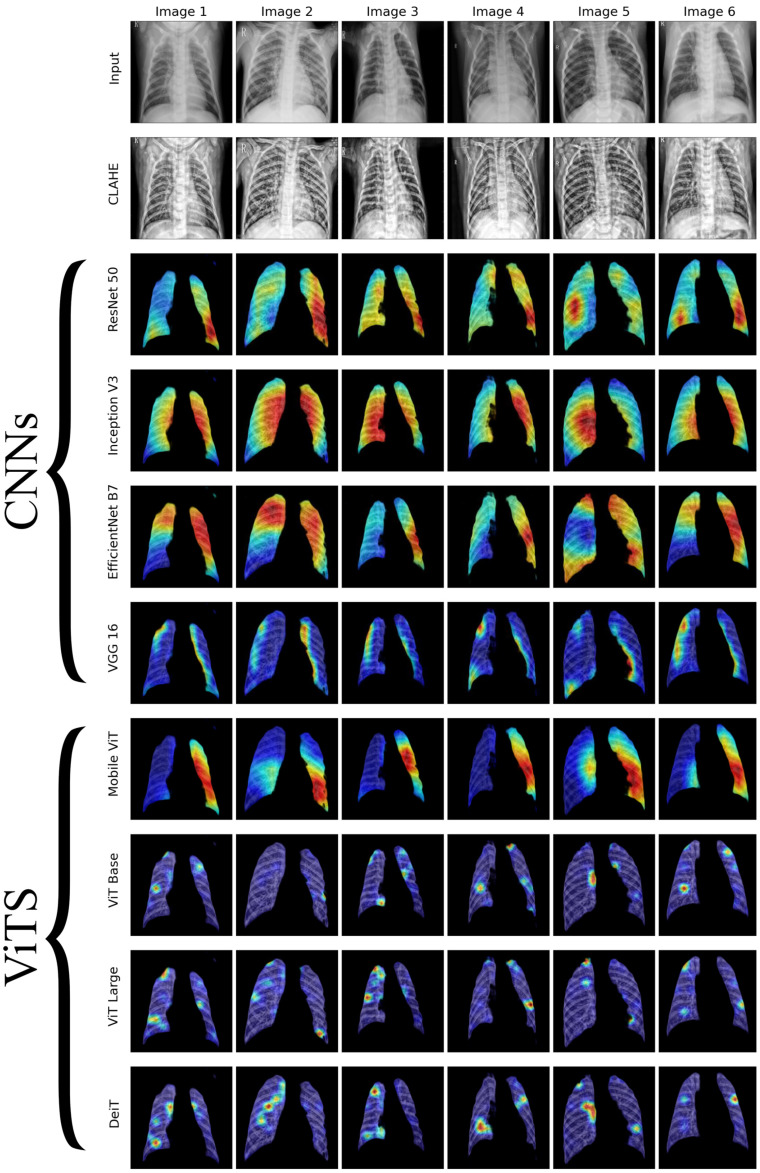
Viral classes heatmaps.

We conducted a thorough examination of several CNN models to identify lung diseases using explainability approaches, particularly heatmaps. This study’s findings reveal the strengths and flaws present in the structures of these models. Each Figure 15, Figure 16, Figure 17, Figure 18, Figure 19, Figure 20, Figure 21 and Figure 22 includes sets with unique images, divided into rows: the first row is input, the second is CLAHE, rows three to six are for CNN results, and rows seven to ten are for ViT results. The EfficientNet models tended to emphasize important characteristics across large portions of the lung. Figure 15 and Figure 16 displays heatmaps for all photos with a widespread presence of red, indicating significant regions. Yet, this model sometimes did not detect any characteristics in the lungs, as seen in Figure 17 (images 3 and 4), indicating imprecision in pinpointing lesions. The Inception V3 model consistently prioritized the center of the images in Figure 15, Figure 16, Figure 17, Figure 18, Figure 19, Figure 20, Figure 21 and Figure 22, showing a tendency towards core image characteristics while perhaps overlooking illness indicators on the periphery. ResNet 50 shows potential by successfully identifying important elements in Figure 15 and Figure 16. However, ResNet 50’s performance varied, as shown in Figure 19 (images 1, 5, and 6) and Figure 18 (all images), where it had challenges in providing explanations, especially for normal class images, indicating reliability issues. VGG 16 exhibited subpar performance in lesion identification. Significant characteristics were often found beyond the segmented lung region, with just the boundaries being highlighted as noteworthy. The mismatch indicates a significant deficiency in VGG 16’s capability to precisely locate lung lesions.

ViT versions, such as ViT-Base, ViT-Large, and the DeiT model, exhibited varied performance. The models demonstrated exceptional accuracy in localizing key characteristics, as seen in Figure 15, Figure 19, Figure 20 and Figure 22. The ViT-Large showed improved ability in detecting lesions, suggesting higher performance in explainability. The MobileViT model demonstrated consistent performance in all categories. The hybrid design, which integrates CNN and transformer components, expanded the scope of significance while reducing interference in masked areas. The enhanced performance of the product was noticeable in Figure 18 and Figure 19, despite certain flaws seen in Figure 21 (image 5) and Figure 15.

The self-attention mechanism enables a detailed comprehension of the spatial relationships among various components of the picture, which aids in a focused examination of the lesions. This is especially advantageous in medical imaging since the differentiation between healthy and pathological tissues may be subtle yet crucial. ViT models enhance the diagnosis of lung lesions by capturing and accentuating specific characteristics, resulting in more exact localization. This improved localization is not only a technological advancement; it signifies a substantial progression in the utilization of DL for medical diagnostics. ViT models excel in identifying and emphasizing numerous distinct lesions with great accuracy, showcasing their promise for the precise diagnosis of lung illnesses, especially in cases when early and accurate identification is crucial. The incorporation of self-attention and localization in ViT models is a significant mark of progress in this area, providing a promising direction for future study and clinical application.

Analyzing DL models for lung illness highlights the significance of explainability in the evaluation and selection process. ViT models, with their improved capacity to concentrate on specific, relevant characteristics, emerge as promising tools in the sector. Their result indicates notable progress in using DL for medical imaging, where precision in identifying disease markers is paramount. Future research should investigate incorporating explainability strategies into model building and clinical operations to connect AI capabilities with healthcare requirements.

## 9. Critical Discussion

This study on DL architectures for lung disease segmentation and classification using CXRs provides a thorough evaluation of several models, highlighting the progress in medical imaging analysis. (I) ViTs have shown better performance than CNNs in capturing global image relationships, which is essential for effectively identifying visually comparable lung diseases. This worldwide viewpoint helps ViTs achieve increased classification accuracy and F1 scores. (II) ViTs stand out by producing more detailed and accurate heatmaps than CNNs, providing doctors with vital insights into the model’s decision-making process by clearly highlighting important characteristics in CXRs. (III) Hybrid models combine ViT’s global contextual awareness with CNNs’ local accuracy to improve generalization capabilities in heatmaps and reliably detect lung lesions while retaining a wide-picture understanding of context. (IV) Attention mechanisms in U-Net have greatly improved compared to standard designs, allowing for a targeted examination of important characteristics and enhancing segmentation accuracy, crucial for discriminating between sick and healthy tissue. (V) Attention U-Nets are superior to regular U-Nest because they use attention processes to adapt to the significance of various parts of an image, resulting in segmentation results that are more accurate and relevant for clinical purposes as Attention U-Nets demonstrated a 24.37% rise in Jaccard index and a 14.31% rise in Dice coefficient from U-Nets. (VI) The effectiveness of AI models in clinical settings is significantly impacted by their explainability. Techniques such as Grad-CAM++ and LPR improve transparency and build confidence among clinicians by graphically explaining the model’s decision-making process. (VII) Although ViTs provide benefits in terms of accuracy and comprehensibility, their implementation in clinical settings is hindered by their high computing requirements, which highlights the need for optimizing the models to enhance their relevance in healthcare. (VIII) Implementing CLAHE as a preprocessing method uniformly improves image quality in various models, such as ViTs and CNNs, highlighting its crucial role in enhancing diagnostic system performance by offering enhanced input data for more precise learning and classification. (IX) An accuracy enhancement of 9% is seen when models are used for segmented image analysis as opposed to raw image classification, as stated in this study. (X) This statistical research showed that ViTs produced statistically significant results, showing that the outcomes were not due to chance. This reinforces our case for using ViTs in clinical and medical sectors.

### 9.1. Benchmarking

Our study outperforms earlier research conducted by Akbulut, Yaman [68], Oh et al. [69], Raza et al. [70], Y.-G. Kim et al. [71], and Alshmrani, Goram et al. [72] in many measures, as shown in Table 6. Our segmentation accuracy, as assessed by the Dice Coefficient, shows strong results, guaranteeing the precise identification of abnormal areas in medical photos. Our Jaccard Index shows a high level of spatial similarity between predicted and ground truth segmentations, highlighting the effectiveness of our technique in reliably capturing key anatomical components. Furthermore, our classification accuracy surpasses that of the majority of comparison research, highlighting the capability of our model to accurately categorize medical photos into different groups. The F1 score, which considers both accuracy and recall, confirms the success of our method in attaining high true positive rates and low false positive rates. Our model is versatile and applicable across many diagnostic settings since it can handle a wide variety of medical classes, as seen by the number of classes it considers. Our proposal prioritizes explainability, while Raza et al. focused on explainability, but their models captured features beyond the lungs. Our method combines LPR and Grad-CAM++ approaches to produce heatmaps that are more precise and appropriate for clinical purposes. The thorough assessment based on accuracy, F1 score, segmentation metrics, class variety, and explainability demonstrates the excellence of our work in enhancing the reliability and practicality of AI systems for medical image processing.

### 9.2. A Special Note on Vision Transformers

ViTs are a major advancement in computer vision that use the transformer architecture from natural language processing (NLP) to evaluate pictures by seeing them as sequences of patches, similar to words in a sentence. This strategy provides substantial benefits. ViTs can analyze the full visual context in one computing step, revealing intricate interconnections between different areas. This is essential for jobs such as medical image classification, when the importance of a feature may be influenced by distant picture regions. ViTs provide great scalability and versatility. They can be easily expanded by adding layers or increasing model size without a corresponding increase in complexity. They are versatile in handling many jobs and may surpass conventional models when provided with enough data and resources. The self-attention mechanism in ViTs offers insights into the model’s decision-making processes. Methods such as Grad-CAM++ and LRP allow for the display of attention patterns, which improves confidence and transparency, especially in fields like medical diagnosis. Although ViTs provide benefits, they encounter challenges such as larger data and computational demands. Methods such as knowledge distillation and model pruning can alleviate these problems. Ongoing research is focused on improving the efficiency of ViT, decreasing data needs, and expanding their range of applications. Ultimately, ViTs provides a novel approach to comprehending visual information by merging overall contextual comprehension with the capacity to provide explanations. ViTs are poised to redefine the limits of AI-enabled vision applications.

### 9.3. Strengths, Weaknesses, and Extensions

This study extensively investigated DL architectures for assessing lung disorders using chest X-rays, focusing on segmentation, classification, and interpretability. The technology accurately forecasts the illness by segmenting the lung, fulfilling regulatory standards and supporting our theory [73]. The research’s main strength is in its thorough comparison of several models including CNNs, ViTs, hybrids, and U-Net with attention mechanisms. This approach highlights the superior accuracy in classifying and segmenting ViTs and attention-enhanced U-Net as compared to traditional CNNs. The significance of interpretability in medical imaging artificial intelligence is highlighted, with technologies like Grad-CAM++ and LRP providing help. This technique may also be used for analyzing long COVID [74]. The effort emphasizes the creation of precise heatmaps to illustrate how AI models make decisions, aiming to enhance trust and encourage the use of AI diagnostics in healthcare environments.

Nevertheless, this research has some limitations. This study’s dependence on a particular dataset and its emphasis on lung disorders might restrict the applicability of the results to other areas of medical imaging or diseases with distinct features. Additionally, the computational needs and resource demands of ViTs might create obstacles for their use in settings with limited resources, which would restrict their availability and broad use in various healthcare contexts.

Future research could concentrate on investigating the scalability of these models on larger and more diverse datasets, including a broader spectrum of lung disorders and perhaps other types of medical imaging techniques [75]. Additionally, exploring methods to improve computational efficiency through the pruning of ViTs might make them more suitable for practical clinical use, especially in settings with limited resources. We can assess several methods for denoising, contrast enhancement, and color normalization [76,77]. Additional research might focus on boosting the interpretability of patient-specific models to provide tailored explanations based on patient contexts, hence improving the possibility for individualized treatment in medical diagnostics using AI. One may attempt using advanced techniques like the Tree Seed Algorithm (TSA) to optimize Artificial Neural Networks (ANNs) for the classification of complex architectural characteristics [78]. Lastly, integrating clinical feedback loops into the AI system architecture may enhance model accuracy and interpretability, allowing AI diagnostics to adapt to new clinical insights and patient outcomes.

## 10. Conclusions

This study highlights significant advancements in medical imaging for identifying lung disorders from CXRs driven by DL technology. ViTs, particularly MobileViT, have demonstrated remarkable performance, achieving an accuracy of 98.52%, which far exceeds the 66.30% accuracy of traditional CNNs like ResNet 50. This illustrates ViTs’ superior capability in analyzing complex global patterns in medical images, where CNNs, limited by their local perspective, are less effective. Additionally, the research underscores the effectiveness of advanced segmentation techniques, specifically the Attention U-Net model, which achieved a Dice Coefficient of 94.24% and a Jaccard Index of 92.43%. The attention mechanism within these models enhances segmentation accuracy by emphasizing essential features, proving crucial in precise medical image analysis. Furthermore, the incorporation of explainability tools such as Grad-CAM++ and LRP provided deeper insights into the decision-making processes of these models. This not only promotes transparency and reliability in AI-based diagnostics but also builds greater trust among medical practitioners. An essential aspect of this study is the critical role of preprocessing methods like CLAHE and image segmentation. These techniques significantly boost model performance, as evidenced by the increase in MobileViT’s accuracy from 90.31% on raw data to 98.52% after preprocessing. This highlights the necessity of high-quality data preparation for training effective models. Overall, the findings set a new standard for the application of ViTs and advanced preprocessing techniques in medical imaging, pointing to a promising path for enhancing diagnostic precision and improving patient outcomes. With ViTs achieving an average accuracy of 95.08% compared to CNNs’ 86.82% accuracy, this research provides a compelling case for the future adoption of these advanced methods in clinical settings.

## Figures and Tables

**Table 1:** Comparison of accuracy, F1-score, recall, and precision between ViTs.

Models	Accuracy (%)	F1-Score (%)	Recall (%)	Precision (%)
MobileViT	98.52	98.50	98.51	98.47
ViT Base	96.84	96.80	97.08	96.71
DEIT	86.84	86.84	87.03	86.69
ViT Large	98.12	98.04	98.15	98.14

**Table 2:** Model metrics on raw image classification.

Models	Accuracy (%)	F1-Score (%)	Recall (%)	Precision (%)
MobileViT	90.31	88.59	87.83	88.59
ViT Base	88.26	89.96	88.20	88.97
DEIT	80.15	77.37	79.88	79.15
ViT Large	88.40	88.21	91.19	90.44

**Table 3:** Comparison of accuracy, F1-score, recall, and precision between all models.

Models	Accuracy (%)	F1-Score (%)	Recall (%)	Precision (%)
ResNet 50	66.30	65.53	66.88	66.74
VGG 16	98.17	98.11	98.00	97.77
Inception V3	93.99	93.54	92.71	94.49
EfficientNet B7	88.84	90.03	90.22	89.62
MobileViT	98.52	98.50	98.51	98.47
ViT Base	96.84	96.80	97.08	96.71
DEIT	86.84	86.84	87.03	86.69
ViT Large	98.12	98.04	98.15	98.14

**Table 4:** Tukey Honestly Significant Difference test.

Group1	Group2	Meandiff	*p*-Value	Lower	Upper	Reject
DeiT	ViT Base	0.0837	<0.001	0.072	0.0953	TRUE
DeiT	ViT Large	0.0921	<0.001	0.0804	0.1037	TRUE
DeiT	MobileViT	0.0677	<0.001	0.056	0.0793	TRUE
ViT Base	ViT Large	0.0084	0.3386	−0.0033	0.02	FALSE
ViT Base	MobileViT	−0.016	<0.001	−0.0277	−0.0043	TRUE
ViT Large	MobileViT	−0.0244	<0.001	−0.0361	−0.0127	TRUE

**Table 5:** McNemar test.

Model Comparison	*p*-Value
DeiT vs. ViT Base	<0.001
DeiT vs. ViT Large	<0.001
DeiT vs. MobileViT	<0.001
ViT Base vs. ViT Large	<0.001
ViT Base vs. MobileViT	<0.001
ViT Large vs. MobileViT	<0.001

**Table 6:** Benchmarking table.

Study	Dice Coefficient	Jaccard Index	Classification Accuracy	F1 Score	Explainability	#Classes
Akbulut, Yaman [68]	×	×	96%	×	×	3
Oh et al. [69]	×	×	97.42%,	×	×	6
Raza et al. [70]	×	×	99.10%	97.28	Grad-CAM	3
Y.-G. Kim et al. [71]	90.8%	×	×	×	×	1
Alshmrani, Goram et al. [72]	×	×	96.48%	95.62%	×	3
Proposed Study	98.54%	97.12%	98.2%	98%	LPR and Grad-CAM++	5

**Figure A1:**
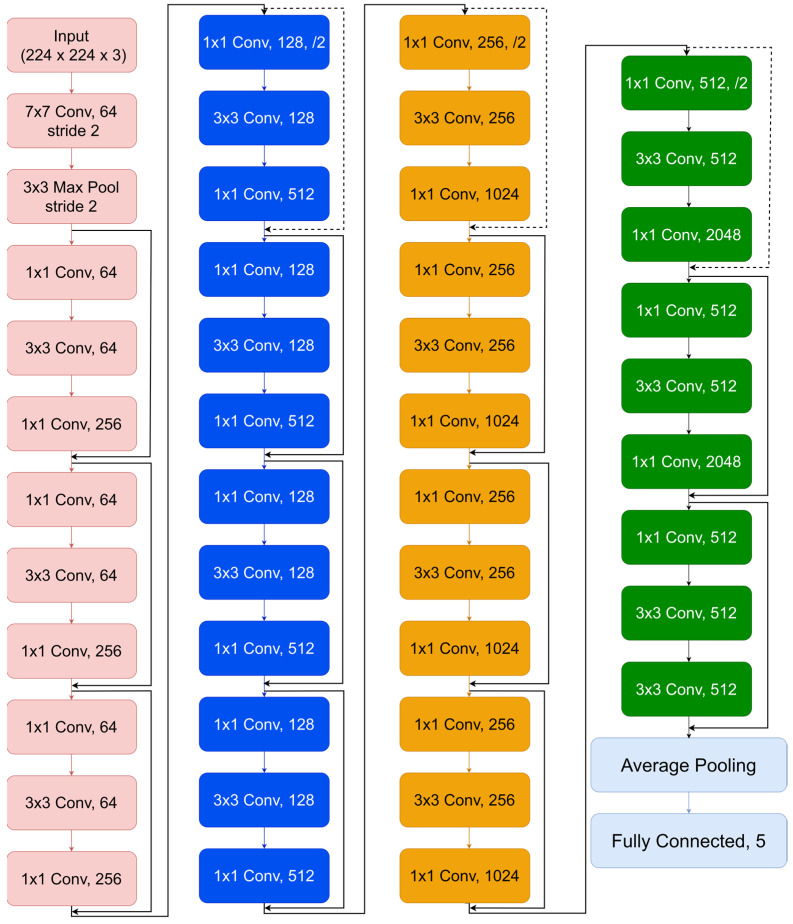
Architecture of ResNet 50 model.

**Figure A2:**
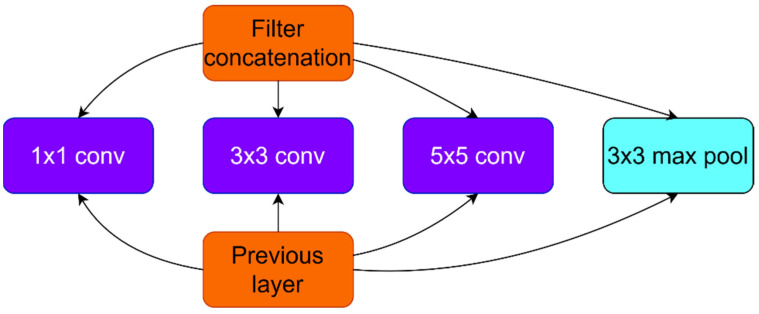
Architecture of Inception module.

**Figure A3:**
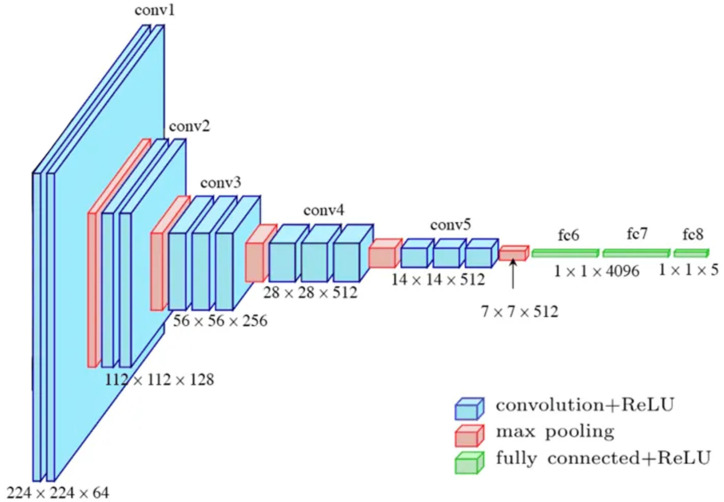
Architecture of VGG 16 model.

## Data Availability

The dataset used in this study can be found in references [46,47,48].

## Appendix A

### Appendix A.1. Conventional Models: Convolutional Neural Networks

Now that we have a deep understanding of Vision Transformers, let us shift our attention to CNNs. CNNs have played a crucial role in image processing for an extended period, providing robust and efficient solutions for various challenges. This section will analyze key CNN architectures, evaluating their operational procedures, advantages, and constraints. This study is crucial for creating a framework to compare the current methodologies of CNNs with the emerging advances in ViTs.

### Appendix A.2. ResNet 50

ResNet is a fundamental deep learning model that enhances model training by the use of residual connections, allowing for the development of more complex network architectures. The proven efficacy of ResNet 50 in various image processing tasks, together with its interpretability features, sets the benchmark for our study, showcasing known CNN methods.

Figure A1 illustrates the ResNet 50 architecture, a deep residual network designed for image classification. The dimensions of the input are 224 pixels in width, 224 pixels in height, and 3 color channels. The input is convolved using a 7 × 7 filter with 64 filters and a stride of 2, followed by max pooling using a 3 × 3 filter with the same stride. This strategy decreases the spatial dimensions and enhances the depth of the feature maps. The heart of ResNet 50 consists of four stages of convolutional blocks, each using a bottleneck design for optimal performance. This design includes a 1 × 1 convolution to decrease dimensionality, a 3 × 3 convolution as the primary feature extractor, and another 1 × 1 convolution to increase dimensionality. Every block has a residual connection that combines the input with the output, helping to train the deep network by addressing the vanishing gradient problem. The network decreases the spatial dimensionality of feature maps by using strides as it progresses through phases. The network’s depth increases, allowing it to learn more complex and abstract information simultaneously. The architecture concludes with average pooling to merge the feature maps into a cohesive global representation, which is then fed into a fully connected layer with five units, aligning with the typical number of output classes seen in our datasets. The ResNet 50 model achieves a favorable equilibrium between depth and computing efficiency, making it a favored choice for advanced image classification tasks.

### Appendix A.3. Inception V3

The Inception V3 model was chosen for its well-constructed convolutional blocks that provide a unique approach to acquiring multi-scale feature representations. The image classification performance of the model has proven exceptional, and its layered interpretability offers crucial insights into the feature extraction mechanisms of CNN models.

Figure A2 depicts a segment of the Inception module, a key element of the Inception network architecture. This module is designed to process input from the previous layer using several convolutional filters of varying sizes and a concurrent max pooling operation. The model employs 1 × 1, 3 × 3, and 5 × 5 convolutions to capture different degrees of spatial context from the input data. The 1 × 1 convolutions decrease dimensionality, enhancing computational efficiency inside the network. The larger 3 × 3 and 5 × 5 convolutions gather more abstract and geographically extensive data. The module includes a 3 × 3 max pooling operation, which provides an extra technique for adjusting features, helping the network to achieve invariance to slight shifts and distortions.

The outcomes of these concurrent procedures are merged across the filter dimension to provide a cohesive output vector that encompasses a broad spectrum of features. The aggregated result is then fed into the subsequent layers of the network. This strategy allows the Inception module to capture representations at various dimensions and degrees of intricacy, enhancing the Inception network’s performance in tasks such as image classification, which deal with objects of diverse sizes and complexity. The Inception design employs modules to provide rapid computation and profound representation, enabling it to outperform competing deep architectures in many vision tasks while using fewer computer resources.

### Appendix A.4. Visual Geometry Group 16

The straightforward architecture of VGG 16, which consists of a series of consistent convolutional layers arranged in a sequential manner, is a basic structure for deep Convolutional Neural Networks (CNNs). VGG 16’s internal representations are readily available for approaches such as Grad-CAM++, making it an excellent model for investigating how model architecture impacts the creation of interpretable visual explanations. Each model in our research was meticulously selected to not only make unique contributions but also provide a thorough analysis of the area of explainable AI in image processing. We wanted to examine a variety of models, ranging from simple to complicated, to comprehend the numerous elements that impact the trade-off between model complexity, performance, interpretability, and computational efficiency. This decision shows our commitment to advancing the AI industry by integrating high-performance standards with the essential aspects of ethical responsibility and transparency in artificial intelligence systems.

Figure A3 illustrates the structure of the VGG 16 model, a convolutional neural network recognized for its simple design and extensive layers. The network processes an image of size 224 × 224 × 3 using convolutional layers (conv1 to conv5) and max pooling layers. Convolutional layers use filters to capture spatial hierarchies of information in images, and the network’s depth allows it to learn complex patterns. Each convolutional layer is followed by a ReLU activation function to introduce non-linearity, allowing the model to learn complex functions. The number of filters in the layers increases from 64 in the first layer to 512 in the final convolutional layers, collecting more complex and abstract information with increased depth. Max pooling layers follow specific convolutional layers shown in red. The layers decrease the size of the feature maps by choosing the greatest value within a restricted region, helping to provide a more compact and translation-invariant representation.

Three fully connected layers (fc6 to fc8) follow the convolutional layers in the architecture. The first two include 4096 channels each, but the third one has 5 channels, which corresponds to the number of classes in datasets such as ImageNet that VGG 16 is often used for. Each fully connected layer is then triggered using a ReLU function. The VGG 16 model is characterized by its uniform architecture, with convolutional layers that use 3 × 3 filters, with a stride of 1 and identical padding, and max pooling layers that use 2 × 2 filters with a stride of 2. The uniformity enhanced the design and significantly contributed to the model’s success in many image recognition tests. VGG 16, despite its simplicity, has a significant amount of parameters, requiring substantial computational resources for training and inference.

### Appendix A.5. EfficientNet B7

EfficientNet B7 is an advanced CNN known for its exceptional accuracy and efficiency in image processing. It utilizes a specialized building component known as MBConv to perform efficiently with reduced computer resource requirements. EfficientNet-B7 stands out for its ability to scale up in a well-proportioned and improved manner. It excels at comprehending visuals without increasing much in size or slowing down. The researchers used an intelligent computer program to optimize the growth of EfficientNet-B7, enhancing its ability to excel in image recognition tasks, as shown by testing.

EfficientNet B7 is a large neural network with 66 million parameters designed to learn from images. Despite its size, this device is designed to operate with high efficiency, enabling it to process intricate images without resource wastage. This is achievable due to its distinctive method of increasing in size, known as “compound scaling,” which maintains equilibrium. The model is adept at learning from diverse images because of its utilization of strategies that enhance flexibility and enable learning from a broad range of photographs. EfficientNet-B7 is a potent tool for image analysis, capable of discerning intricate details and deriving insights in an intelligent and efficient manner.
